# Role of CD4^+^ T-cells for regulating splenic myelopoiesis and monocyte differentiation after experimental myocardial infarction

**DOI:** 10.1007/s00395-024-01035-3

**Published:** 2024-03-04

**Authors:** Nadine Gladow, Claudia Hollmann, Johannes Weirather, Xin Ding, Matthias Burkard, Sabrina Uehlein, Richa Bharti, Konrad Förstner, Thomas Kerkau, Niklas Beyersdorf, Stefan Frantz, Gustavo Ramos, Ulrich Hofmann

**Affiliations:** 1 Department of Internal Medicine I, University Clinic Würzburg, Würzburg, Germany; 2 Comprehensive Heart Failure Centre, University Clinic Würzburg, Würzburg, Germany; 3 Institute for Virology and Immunobiology, University of Würzburg, Würzburg, Germany; 4 MorphoSys AG, Planegg, Germany; 5 Present Address: Department of Cardiology and Angiology, Hannover Medical School, Hannover, Germany; 6 TUM Campus, Straubing for Biotechnology and Sustainability, Weihenstephan-Triesdorf University of Applied Sciences, Straubing, Germany; 7 ZB MED-Information Centre for Life Sciences, Cologne, Germany; 8 Faculty of Information Science and Communication Studies, Cologne University of Applied Sciences, Cologne, Germany

**Keywords:** Myocardial infarction, Monocytes, Lymphocytes, Treg

## Abstract

Myocardial infarction (MI) induces the generation of proinflammatory Ly6C^high^ monocytes in the spleen and the recruitment of these cells to the myocardium. CD4^+^ Foxp3^+^ CD25^+^ T-cells (Tregs) promote the healing process after myocardial infarction by engendering a pro-healing differentiation state in myocardial monocyte-derived macrophages. We aimed to study the effects of CD4^+^ T-cells on splenic myelopoiesis and monocyte differentiation. We instigated MI in mice and found that MI-induced splenic myelopoiesis is abrogated in CD4^+^ T-cell deficient animals. Conventional CD4^+^ T-cells promoted myelopoiesis in vitro by cell–cell-contact and paracrine mechanisms, including interferon-gamma (IFN-γ) signalling. Depletion of regulatory T-cells enhanced myelopoiesis in vivo, as evidenced by increases in progenitor cell numbers and proliferative activity in the spleen 5 days after MI. The frequency of CD4^+^ T-cells-producing factors that promote myelopoiesis increased within the spleen of Treg-depleted mice. Moreover, depletion of Tregs caused a proinflammatory bias in splenic Ly6C^high^ monocytes, which showed predominantly upregulated expression of IFN-γ responsive genes after MI. Our results indicate that conventional CD4^+^ T-cells promote and Tregs attenuate splenic myelopoiesis and proinflammatory differentiation of monocytes.

**Supplementary Information:**

The online version contains supplementary material available at 10.1007/s00395-024-01035-3.

## Introduction

Monocyte-derived cells are centrally involved in all stages of ischaemic heart disease. During atherogenesis, monocyte accumulation in the vessel wall feeds the macrophage and foam cell pools, thereby contributing to vessel inflammation and disease progression [21, 22]. Ischaemia following atherosclerotic plaque rupture and thrombotic coronary artery occlusion inflicts a cardiac wound that triggers the inflammatory pathways and thus leads to the rapid recruitment of myeloid cells from the circulation into the myocardium. Monocyte-derived macrophages are centrally involved in resorption of necrotic myocardial tissue, resolution of inflammation during post-infarction healing, and chronic remodelling of the left ventricle [9]. Although monocytes are integral components of cardiac healing [8], an unbalanced or exaggerated immune reaction after myocardial infarction (MI) aggravates the tissue damage that triggers maladaptive remodelling [7]. Therefore, there is a clinical need to gain deeper insight into the factors regulating monocyte production and differentiation after MI.

Early after MI, the monocyte reservoir in the splenic red pulp supplies a large proportion of monocytes to the infarct zone [12, 20, 28]. The importance of the splenic-monocyte pool to proper early healing is underscored by the demonstration, in mice, that splenectomy during the first week after MI drastically reduced monocyte numbers in the myocardium and compromised infarct healing [12, 20]. To meet the continuously high demand for monocytes during the entire healing process, an “emergency myelopoiesis” programme starts in the spleen [6]. This allows for continual recruitment of splenic monocytes to the healing myocardium. Mechanistically, the initiation of sympathetic activity after MI and the subsequent noradrenaline release leads to the engagement of beta- adrenergic receptors on bone marrow stromal cells, resulting in the downregulation of stem cell retention factors, such as CXCL-12 or vascular cell adhesion molecule 1 (VCAM-1). As a consequence of this process, haematopoietic stem cells (HSCs) are liberated from the bone marrow into the circulation [4, 5]. Released HSCs seed into the spleen where they expand in situ and undergo myeloid lineage commitment and progenitor cell differentiation [4–6, 20].

Like the innate immune system, the adaptive immune system also activates in response to MI, and CD4^+^ Foxp3^+^ CD25^+^ T-cells (regulatory T-cells; Tregs) have been shown to pivotally facilitate cardiac healing [2, 14, 15]. Tregs crucially improve post-infarction healing by mitigating cardiac inflammation [26]. Moreover, Tregs can modulate fibroblast function and programme macrophage polarisation in the infarct zone towards a differentiation state that promotes healing [26, 30]. However, the infarct zone contains substantially more monocytes than CD4^+^ T-cells, which suggests CD4^+^ Tregs may exert their healing supportive function, at least partially, outside the infarcted heart.

In addition to multi-organ autoimmunity, mice with a constitutive loss-of-function mutation in the Foxp3 gene show spontaneous myelopoiesis due to losing the inhibition of the lymphocytes that produce myelopoiesis-inducing proteins in the spleen [18, 27]. In the absence of Treg-mediated suppression, granulocyte–monocyte colony stimulating factor (GM-CSF) production by CD4^+^ T-cells induces splenic but not bone marrow myelopoiesis. This indicates that Tregs are necessary to counter-regulate spontaneous splenic myelopoiesis in the steady state. Nevertheless, it is not yet known whether Tregs also regulate extramedullary myelopoiesis in response to sterile tissue injury. This prompted us to hypothesise that in addition to their known effect on monocyte recruitment and their local differentiation in the infarcted myocardium, Tregs may also regulate monocyte production and differentiation in the spleen. Therefore, we studied the impact of Tregs and Tconv on splenic myelopoiesis and monocyte differentiation in response to experimental MI.

## Methods and materials

### Mice and surgeries

Male mice between 8 and 10 weeks of age were used for all experiments. Mice expressing the human diphtheria toxin receptor under the control of the Foxp3 promotor, named B6.129(Cg)*Foxp3*^*tm3Ayr*^/J (Foxp3^DTR^ mice; RRID:IMSR_JAX:016958), and the respective wild type (WT) controls from Jackson Laboratory were used for pharmacological ablation of CD4^+^ Foxp3^+^ T-cells.

Myocardial infarction was induced by permanent ligation of the left anterior descending coronary artery, as described previously [10]. Mice were anaesthetized using 3–4% isoflurane, intubated, and ventilated with oxygen/0.8–1.2% isoflurane. Permanent MI was induced as described previously [16]. For the sham surgery, a thoracotomy was performed without ligating the vessel. All mice undergoing surgeries were injected with buprenorphine (0.1 mg/kg intraperitoneally) for analgesia. In the Treg cell ablation experiments, 500 ng diphtheria toxin (DTX, Merck Millipore) per mouse was injected intraperitoneally on day 2 and day 1 before surgery. To prevent Treg cell rebound, 250 ng DTX per mouse was in addition injected on day 2 after the MI or sham operation.

### Flow cytometry

To stain mature leukocytes from the lymph nodes and spleen, single-cell suspensions were prepared. Up to 10^6^ cells were stained in 50 µl PBS + 0.5% BSA + 0.05% NaN_3_ + 1 mM EDTA. Following Fc receptor blockade with the antibody clone 2.4G2 (20 min, 4 °C), fluorochrome-labelled antibodies against surface leukocyte markers were added. To stain splenic stem and progenitor cells, splenocytes were resuspended in BSS + 5% BSA. B-cells, T-cells, and erythrocytes were depleted from the suspension using magnetic separation according to the manufacturer’s protocol (Miltenyi). In brief, after Fc receptor blockade, biotinylated antibodies against TCR-β, CD19, and Ter119 were added to the cell suspension. After 15 min of incubation on ice, the cells were washed in BSS + 5% BSA and resuspended in BSS + 5% BSA. Streptavidin-coated beads were added. After incubation, the cells were washed and applied to an LS-MACS column (Miltenyi). The flow-through was used for surface marker labelling. The following antibodies (clones) were used: anti-CD11c (N418), anti-CD127 (A7R34), anti-CD25 (PC61), anti-CD19 (6D5) anti-CD3 (17A2), anti-CD45 (30-F11), anti-CD4 (RM4-5), anti-CD49b (DX5), anti-FceR1α (MAR-1), anti-CD117 (2B8), anti-Ly6G (1A8), anti-CD34 (HM34), anti-NK1.1 (PK136), anti-Sca-1 (D7), anti-CD48 (HM48-1), anti-CD150 (TC15-12F12.2), anti-CD11b (M1/70), anti-CD135 (A2F10), anti-Ly6C (HK1.4), anti-CD115 (AFS98), anti-CD8 (53–6.7), and anti-B220 (RA3-6B2). The gating strategy and progenitor-subset definitions are depicted in Supplementary Fig. 4.

For intracellular cytokine/growth factor staining, cells were stimulated ex vivo for 6 h (37 °C) in RPMI 1640 + 10% FBS with phorbol-12-myristate-13-acetate (PMA, Sigma-Aldrich) and ionomycin (Sigma-Aldrich) in the presence of monensin (BioLegend). For intracellular staining with anti-GM-CSF (MP1-22E9), anti-IFN-γ (XMG1.2), anti-IL-3 (MP2-8F8), anti-IL-6 (MP5-20F3), or anti-Foxp3 (FJK 16 s) antibodies, cells were fixed, permeabilized (with fixation/permeabilization solution; BD Bioscience according to the manufacturer’s protocol), and stained with the appropriate antibodies (all purchased either from BD Biosciences or BioLegend). To quantify the splenic stem and progenitor cells, counting beads (Thermo Fisher Scientific) were included. Cells were analysed on an LSR II flow cytometer (BD Biosciences) with FlowJo software (TreeStarInc; RRID:SSR_008520). Cell sorting was performed using a FACSAria III (BD Biosciences).

### In vivo proliferation of stem and progenitor cells

To assess the proliferation of stem and progenitor cells in vivo, mice were injected with 50 mg/kg EdU intraperitoneally 1 h before euthanasia. Prior to staining with surface antibodies, single-cell suspensions were stained with EdU by using a Click-iT EdU Alexa Fluor 488 Kit (Thermo Fisher Scientific) according to the manufacturer’s instructions. In brief, cells were incubated with 50 µl Click-iT^®^ fixative for 15 min at room temperature. After washing and permeabilizing, cells were incubated for 30 min with the Click-iT reaction cocktail containing the fluorescent dye, azide, and CuSO_4_. After washing, the resuspended cells were analysed on a flow cytometer.

### In vitro studies

CD4^+^ CD25^+^ regulatory T-cells and CD4^+^ CD25^−^ T-cells were isolated from C57BL/6 J mice with the magnetic-activated cell sorting kit from Miltenyi (CD4^+^ CD25^+^ Regulatory T Cell Isolation Kit). The cells were stimulated with CD3/CD28 (Dynabeads Mouse T-Activator CD3/CD28 from Gibco) for 24 h. For coculture experiments, these stimulated CD4^+^ T-cells were cultured together with isolated CFSE-labelled bone marrow cells from CD4KO mice (B6.129S2-*Cd4*^*tm1Mak*^/J from Jackson Laboratories) in the presence or absence of serum from 5 day MI or sham-operated WT animals. The in vitro studies were performed in RPMI medium supplemented with 10% FBS for 72 h at 37 °C. For transwell assays, membranes (Corning Life Sciences) with 0.4 µm pores were used to separate Tregs or Tconv cells from bone marrow cells. 100U/ml IFN-gamma (Peprotech) were added to the cell culture when indicated.

### Quantitative real-time PCR

Messenger RNA (mRNA) was extracted from the spleens of non-reperfused mice using a RNeasy Mini Kit (Qiagen) according to the manufacturer’s instructions. One microgram of mRNA was transcribed to complementary DNA (cDNA) with an iScript kit (Bio-Rad). TaqMan primers (Life Technologies) were employed for quantitative real-time PCR (Applied Biosystems). The results are expressed as Ct values normalised to the housekeeping gene Gapdh. For real-time PCR analysis of FACS-sorted cells, a RNeasy Micro Kit (Qiagen) was applied, and the mRNA was amplified using a REPLI-g WTA Single Cell kit (Qiagen) according to the manufacturer’s protocol.

### Transcriptome analysis

Splenic monocytes were isolated by fluorescence activated cell sorting (FACS) as CD11b^+^ Ly6G^−^ F4/80^+^ Ly6C^hi^ CD115^+^ cells. Samples of 6800–26,800 Ly6C^hi^ splenic monocytes were collected directly into 150 μl RLT lysis buffer (containing 10% β-mercaptoethanol). RNA extraction was performed using a RNeasy Mini Kit (Qiagen) according to the manufacturer’s instructions. The quantity and quality of the extracted RNA were tested with an Agilent 2100 Bioanalyzer RNA Pico assay (Agilent Technologies). An Ovation SoLo RNA-Seq Mouse System (NuGEN) was used for library preparation, following the manufacturer’s instructions, starting with 20–50 pg good-quality RNA (RIN > 7) as the input. The final libraries were quantified using a Qubit 2.0 Fluorometer (Invitrogen) and tested with an Agilent 2100 Bioanalyzer RNA Nano assay (Agilent Technologies). The libraries were then processed on an Illumina cBot for cluster generation on the flowcell, following the manufacturer’s instructions, and 30 million reads were sequenced in the single-end 50 bp mode on a HiSeq2500 (Illumina). The CASAVA 1.8.2 version of the Illumina pipeline was used to process the raw data for both format conversion and de-multiplexing.

The raw reads were processed using a FastQC 0.11.3 to assess read quality, number of duplicates, and adapter sequences. The Illumina TruSeq adaptors were removed using Cutadapt software (version 1.12) and the reads were further trimmed to maintain a quality drop value below a mean of Q20. Following this, the processed sequences were mapped to the murine genome (mm12, GRCm38) using the short read aligner STAR-2.5.2b software [3] with genome and annotation files retrieved from GENCODE. For all studied samples, the proportion of reads mapped to the mouse reference genome ranged between 78 and 83%. The reads aligning to specific genes were quantified using BEDTools software [24], subcommand intersect (version 2.15.0). Differentially expressed genes were identified using DESeq2 software (version 1.16.1). The genes with a Benjamini–Hochberg corrected *P* value below 0.05 were classified as significantly differentially expressed. Heat maps were used to represent the top 50 genes differentially expressed by log_2_-fold-change. In addition, gene ontology (GO) term enrichment analyses for differentially expressed genes were carried out using Innate DB database web service [1]. In each case, only the GO terms with a corrected *P* value below 0.01 were reported and visualised.

Protein–protein interaction analysis was performed using the STRING database (http://string-db.org/).

### Statistics

Statistical analyses were performed using GraphPad Prism version 5 software (RRID:SCR_002798). The results are displayed as the mean ± standard deviation. For two-group comparisons, an unpaired t test was conducted for normally distributed variables. For multiple comparisons, 2-way ANOVA was used. *P* values < 0.05 were considered statistically significant.

## Results

### Changes in the splenic CD4^+^ T-cell compartment after myocardial infarction

During the first week after MI, the spleen is the major source for monocytes recruited to the myocardium. To replenish the splenic pool splenic myelopoiesis peaks around day 5 [20]. Accordingly, when comparing cell numbers in sham vs MI animals, we found significantly increased monocyte progenitor cells (HSCs and GMPs/CMPs) at day 5 in the spleen but not in the bone marrow (Suppl. Figure 1a,b). Furthermore, the frequencies of EdU^+^ HSCs, MPPs and GMP/CMPs were significantly higher in the spleens of MI vs sham-operated animals, whereas in the bone marrow the proliferative activity of progenitor cells was unchanged (Suppl. Figure 1c). Moreover, 56 days after MI there were no differences in progenitor cells between sham and MI animals within the spleen and the bone marrow (Suppl. Figure 1a,b).

**Fig. 1:**
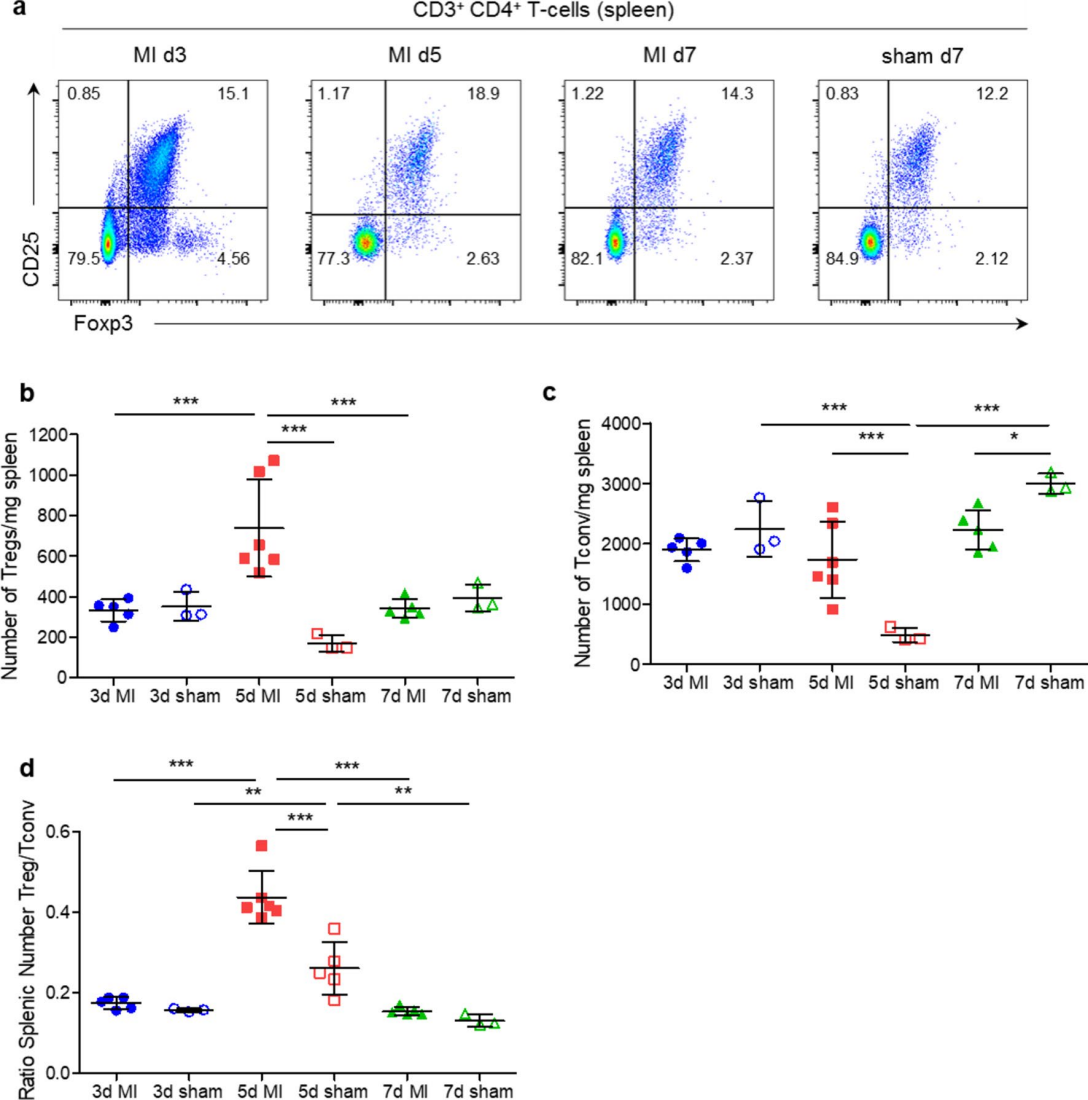
CD4^+^ T-cell subsets in the spleen. **a** Flow cytometry plots showing CD25 and Foxp3 expression in splenic CD4^+^ T-cells. **b**, **c** Absolute numbers of CD4^+^ Foxp3^+^ CD25^+^ (Treg; b) and CD4^+^ Foxp3^−^ (Tconv; c) T-cells in the spleens of post-MI and sham-operated animals on days 3, 5, and 7 after surgery. **d** Ratio of splenic Treg/Tconv numbers in the infarcted and sham-operated mice 3, 5, and 7 days after sham or MI surgery. *MI* myocardial infarction. Data are presented as the mean ± SD. *P < 0.05, **P < 0.01, ***P < 0.001 (**b-d** two-way ANOVA)

As CD4^+^ T-cells might play a role in regulating MI-induced splenic-monocyte production, we analysed the composition of the splenic CD4^+^ T-cell compartment, including both regulatory (Treg: Foxp3^+^ CD25^+^) and conventional (Tconv: Foxp3^−^) T-cells, within the first week after MI (Fig. 1a). The absolute Treg counts on day 5 are significantly higher than on days 3 and 7 after MI. Moreover, there are increased Treg numbers in MI vs. sham on day 5 (Fig. 1b). In contrast, the absolute number of splenic Tconv did not change between days 3, 5, and 7 after MI (Fig. 1c). The Treg:Tconv ratio in the spleen was significantly higher on day 5 when comparing sham vs. MI and compared to day 3 or 7 after MI (Fig. 1d). In comparison to the spleen, no changes in Treg:Tconv ratio between sham and MI were detected in the bone marrow 5 days after surgery (Suppl. Figure 2f). Notably, the frequencies of B- and T-cells are significantly lower in the bone marrow and do not change after MI (Suppl. Figure 2a-e). As these transient changes in CD4^+^ T-cell composition could affect myelopoietic activity in response to MI, we further studied the role of Tconv and Tregs for myelopoiesis.

**Fig. 2:**
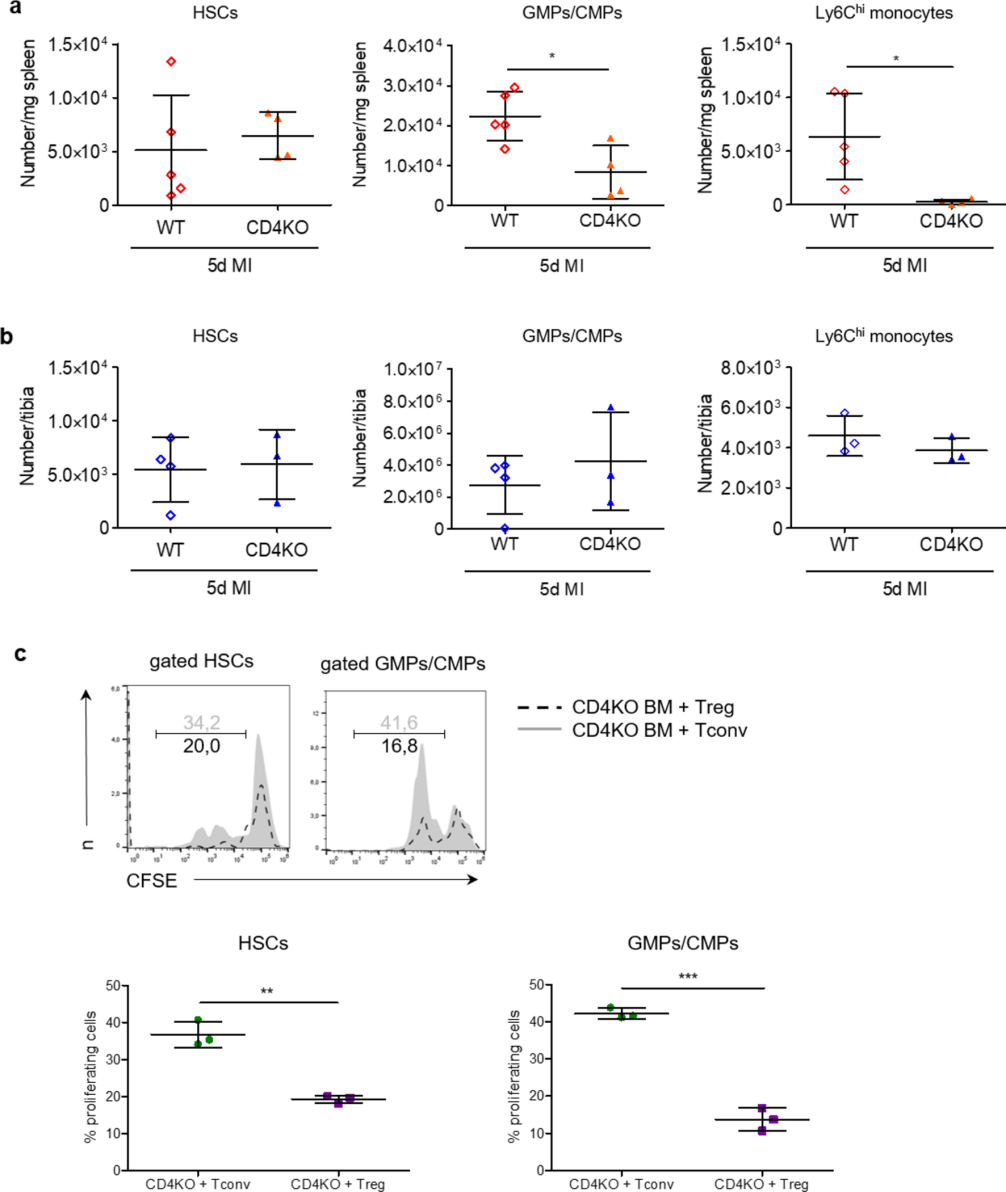
Role of CD4^+^ T-cells in splenic and bone marrow myelopoiesis. Quantification of HSCs, GMPs/CMPs, and monocytes in the spleens **a** and bone marrow **b** of WT and CD4 knockout mice (CD4KO) 5 days after MI. **c** Quantification of proliferative activity of HSCs and GMPs/CMPs isolated from CD4KO BM after coculture with splenic Tregs or Tconv from WT mice. *HSC* haematopoietic stem cell, *GMP* granulocyte–macrophage progenitor, *CMP* common myeloid progenitor. Data are presented as the mean ± SD. *P < 0.05, **P < 0.01, ***P < 0.001 (*t*-test)

To elucidate the role CD4^+^ T-cells play in splenic myelopoiesis after MI, we analysed myelopoietic activity in CD4 KO animals vs. WT animals 5 days after MI. We found significantly lower numbers of both myeloid progenitor cells and Ly6C^high^ monocytes in the spleens of CD4 KO mice 5 days after MI (Fig. 2a). In contrast to the spleen, no significant differences in progenitor cell numbers and monocytes in the bone marrow of WT and CD4KO mice were found after MI (Fig. 2b). CD4 KO mice show adverse healing with enhanced inflammation in the heart, which might remotely interfere with splenic myelopoiesis [14]. To dissect the impacts of conventional vs. regulatory CD4^+^ T-cells on myelopoiesis post MI independently from systemic effects caused by different levels of myocardial injury and inflammation, we cultured bone marrow cells from CD4KO mice harvested 5 days after MI. Adding Treg cells did not affect ex vivo proliferation of hematopoietic precursor cells harvested from CD4 KO animals 5 days post MI. In contrast, coculture with activated Tconv enhanced proliferation of precursor cell populations (including HSCs and GMP/CMPs, Fig. 2c). Hence, these results indicate that CD4^+^ T-cells are required for splenic myelopoiesis in response to MI. Tconv promote myelopoiesis, whereas Treg have no impact in the absence of Tconv.

### Regulatory T-cell depletion enhances splenic myelopoiesis after myocardial infarction

**Fig. 3:**
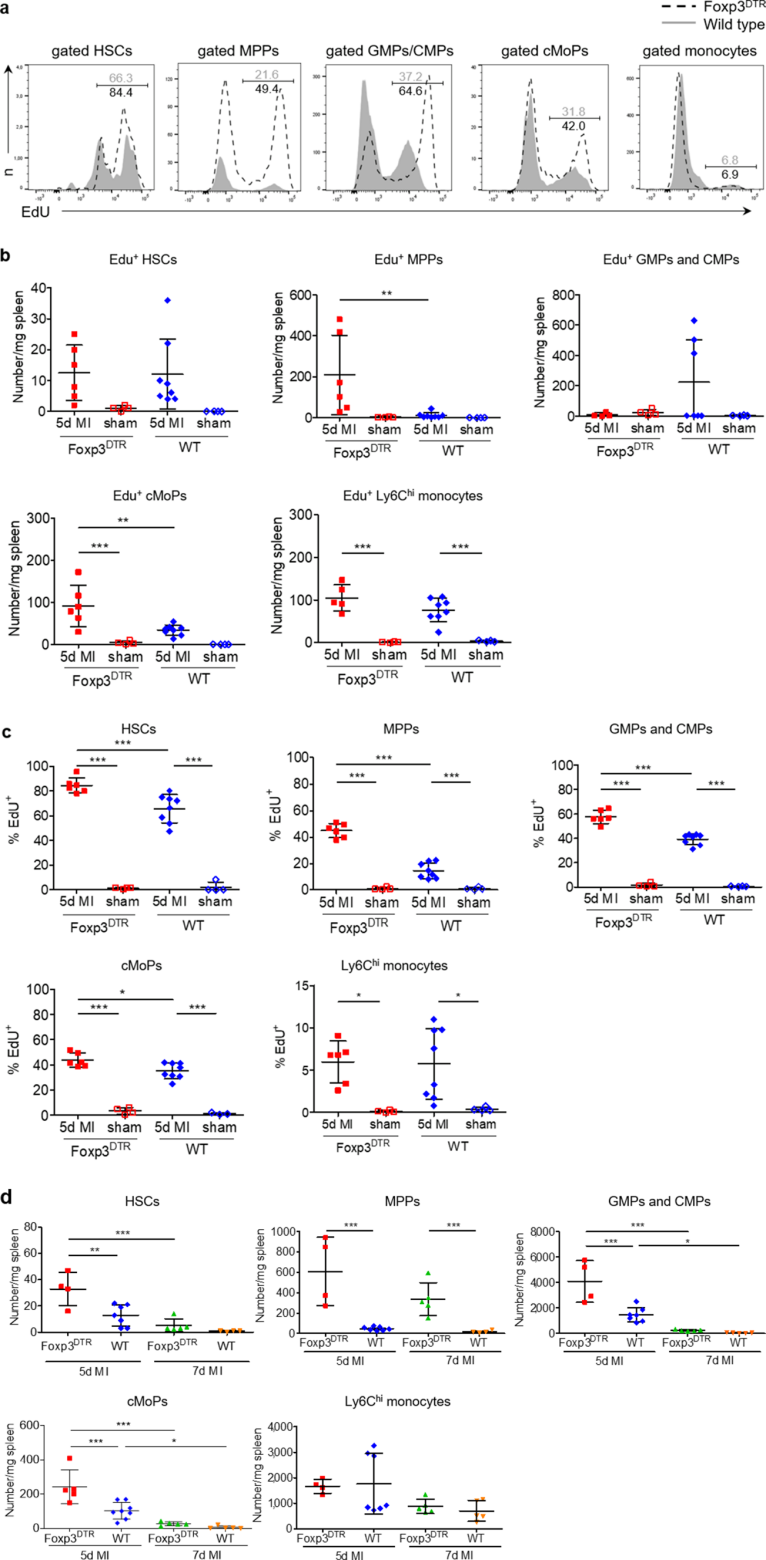
Proliferation analysis and quantification of precursor cells and monocytes in wild type (WT) and Foxp3^DTR^ mice after myocardial infarction. **a**, **b**, **c** In vivo proliferation of HSCs, MPPs, GMPs/CMPs, cMoPs, and monocytes in the spleen 5 days after MI in wild type vs. Foxp3^DTR^ mice. Representative plots **a** and quantitative data **b**, **c** for intracellular EdU analysis in MI animals. **d** Quantification of HSCs, MPPs, GMPs/CMPs, cMoPs, and monocytes in the spleens of WT and Treg-depleted mice (Foxp3^DTR^) 5 and 7 days after MI. *HSC* haematopoietic stem cell, *MPP* multipotent progenitor; *GMP* granulocyte–macrophage progenitor, *CMP* common myeloid progenitor, *cMoP* common monocyte progenitor. Data are presented as the mean ± SD. *P < 0.05, **P < 0.01, ***P < 0.001 (**b**, **c**, **d** two-way ANOVA)

To further study the effect of Tregs on splenic myelopoiesis after MI in vivo, we analysed progenitor cell numbers in spleens from WT and Foxp3^DTR^ mice, both treated with diphtheria toxin to selectively deplete Tregs in Foxp3^DTR^ mice (Fig. 3). As we recently showed, this specific Foxp3^+^ cell depletion approach impairs the clinical outcome after MI by inducing a proinflammatory differentiation state in myocardial macrophages [15]. Notably, our protocol resulted in approximately 60% fewer Foxp3^+^ cells within the total CD4^+^ T-cell populations in the secondary lymphoid organs, including the spleen, when comparing Foxp3^DTR^ mice with WT mice (Suppl. Figure 3). Lineage^−^ Sca-1^+^ CD117^+^ cell subsets resembling HSCs supplied by the bone marrow and multipotent haematopoietic progenitors (MPPs) and Lineage^−^ CD117^±^ myeloid progenitor cell subsets, including common myeloid progenitors (CMPs), granulocyte–monocyte progenitors (GMPs), common monocyte progenitors (cMoPs), and monocytes, were analysed in the spleen by flow cytometry (Suppl. Figure 4). We studied cellular proliferative activity in vivo by applying the nucleoside analogue 5-ethynyl-2’-deoxyuridine (EdU; Fig. 3a). The absolute number of EdU^+^ monocytes (Fig. 3b) and the frequency of EdU^+^ HSCs, MPPs, GMPs/CMPs, cMoPs, and monocytes (Fig. 3c) were significantly higher in infarcted WT mice than in sham-operated WT animals. After MI, the numbers of EdU^+^ MPPs and cMoPs and the frequencies of EdU^+^ HSCs, MPPs, GMPs/CMPs, and cMoPs were significantly higher in Foxp3^DTR^ mice than in control WT mice (Fig. 3b, c), further demonstrating the increased in vivo proliferation of progenitor/stem cells in response to infarction in Treg-depleted animals. In the absence of MI, the Foxp3^+^ T-cell ablation approach per se did not considerably impact splenic progenitor cell proliferation. There was no significant difference in the frequency of EdU^+^ stem/ progenitor cells between sham-operated WT mice and Foxp3^DTR^ mice on day 5 (Fig. 3c).

Moreover, the absolute numbers of all hematopoietic stem/ progenitor cell populations per spleen were higher on day 5 than on day 7 after MI in WT mice (Fig. 3d). In Foxp3^DTR^ mice, the total numbers of HSC, MPPs, GMPs/CMPs, and cMoPs were significantly elevated compared to those of WT mice at day 5 after MI (Fig. 3d, Suppl. Figure 5). The number of Ly6C^high^ monocytes in the spleen of Foxp3^DTR^ mice remained unchanged compared to that in WT animals after MI (Fig. 3d). However, Ly6C^high^ monocytes in diphtheria toxin-treated Foxp3^DTR^ were more numerous in the infarct and border zones of the myocardium and in the blood, compared to those in diphtheria toxin-treated WT mice (Suppl. Figure 6). These comparisons indicate that the increased splenic myelopoietic activity in response to Treg depletion feeds the myocardial monocyte supply, leading to increased proinflammatory monocyte numbers in the myocardium.

### Tregs suppress production of the cytokines that drive splenic myelopoiesis

Next, we explored whether Tconv in the spleen express cytokines, known to promote myelopoiesis. We detected a transient, modest but significant upregulation of GM-CSF expression in the spleen following MI. GM-CSF expression in the spleen was notably higher in MI vs. sham-operated animals on day 5 (Suppl. Figure 7a). Small subsets of T-cells and B-cells stained positive for intracellular GM-CSF (Suppl. Figure 8a, b) in the spleen, albeit with no difference between the sham-operated and post-MI groups. In addition, we analysed gene expression of other factors that might be involved in splenic Tconv and myelopoiesis crosstalk, including CXCL-12, IL-7, IL-23, IL-2, and IL-1β (Suppl. Figure 7b-f). None of these showed any significantly altered expression in splenic tissue from either sham or MI.

**Fig. 4:**
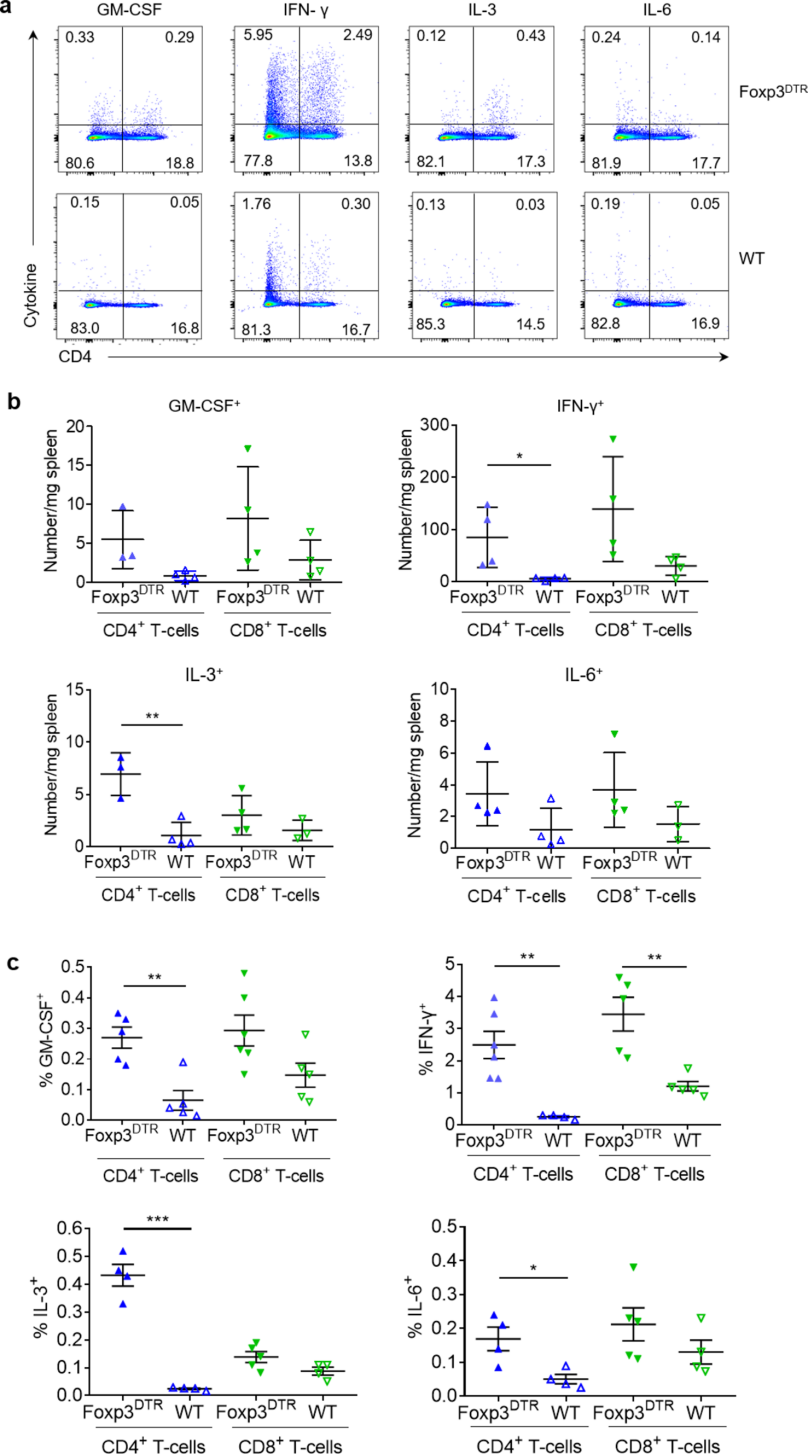
Cytokine expression in the spleen 7 days after myocardial infarction in Treg-depleted (Foxp3^DTR^) and wild type (WT) mice. **a** Representative flow cytometry plots showing intracellular cytokine expression in CD4^+^ T-cells of Foxp3^DTR^ and wild type mice 7 days after MI. **b**, **c** Expression of GM-CSF, IFN-γ, IL-3, and IL-6 in CD4^+^ and CD8^+^ T-cells in the spleens of Foxp3^DTR^ and wild type mice 7 days after MI. **c**, **d** Data are shown as the mean ± SD. *P < 0.05, **P < 0.01, ***P < 0.001 (*t*-test)

To better understand how Treg–Tconv interaction after MI modulates myelopoiesis, we compared the intracellular expression of those proteins in lymphocytes from WT and Foxp3^DTR^ mice after MI (Fig. 4a). We detected significant increase in the numbers of both IFN-γ- and IL-3-expressing CD4^+^ T-cells in the spleens of Foxp3^DTR^ mice after MI (Fig. 4b). Furthermore, Foxp3^+^ T-cell depleted Foxp3^DTR^ mice showed more frequent IFN-γ^+^ CD4^+^ and CD8^+^ T-cells and significantly more frequent IL-3-, GM-CSF, and IL-6-expressing CD4^+^ T-cells (Fig. 4c). These results indicate that Tregs attenuate the production of several myelopoiesis-promoting cytokines in T-cells.

### Treg suppression of splenic myelopoiesis depends on cell-contact and IFN-γ

**Fig. 5:**
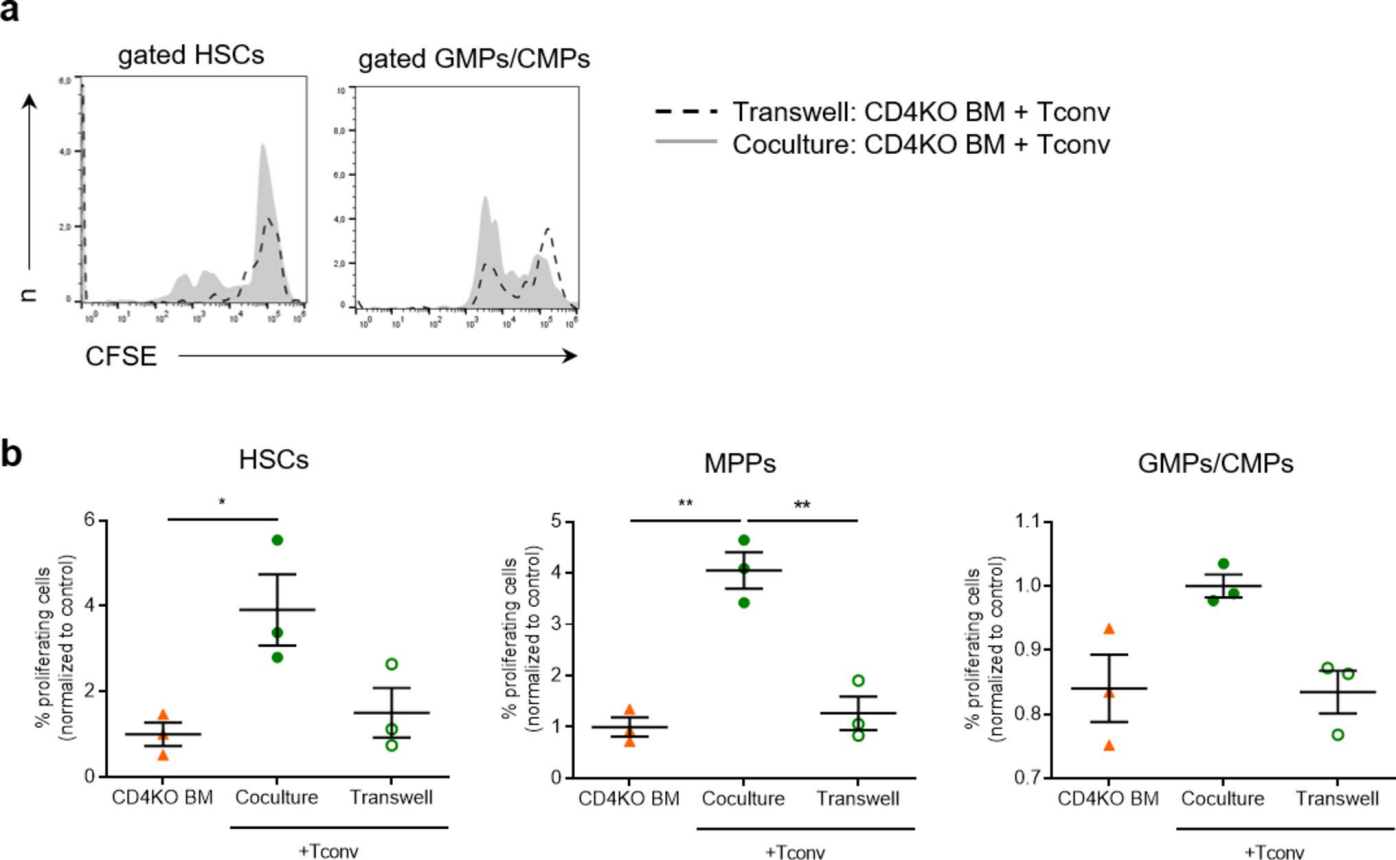
Role of Tconv in progenitor cell proliferation in vitro. **a** Representative flow cytometry plots showing in vitro proliferation of HSCs and GMPs/CMPs from the BM of CD4KO mice in a coculture vs. transwell system with splenic Tconv from WT animals. **b** The proliferative activity of CD4KO bone marrow precursor cell populations was measured by CFSE staining and normalized to CD4KO BM cultured without splenic Tconv cells from WT animals. Data are presented as the mean ± SD. *P < 0.05, **P < 0.01 (one-way ANOVA)

**Fig. 6:**
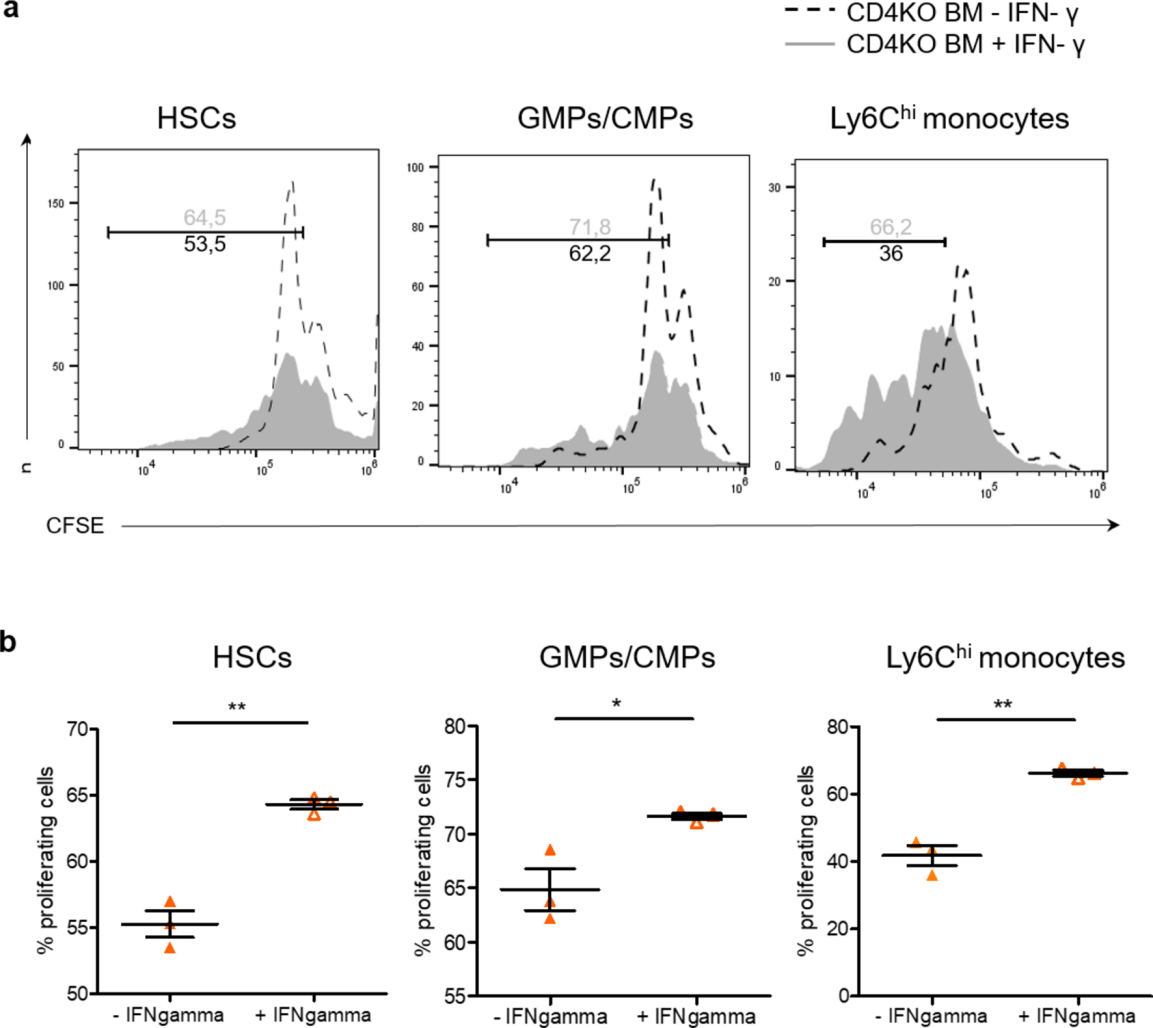
Effect of IFN-gamma on progenitor cell proliferation in vitro. **a** Representative flow cytometry plots showing the proliferative activity of HSCs, GMPs/CMPs, and monocytes, isolated from CD4KO BM, in the presence or absence of IFN-γ. **b** The proliferative activity was measured by CFSE staining. Data are presented as the mean ± SD. *P < 0.05, **P < 0.01(*t*-test)

To further mechanistically dissect if Tregs limit myelopoiesis by interacting with Tconvc or through direct effects on progenitor cells, we performed transwell cell culture assays with bone marrow cells from CD4 KO donors. We found HSCs, MPPs, and GMPs/CMPs from CD4 KO donors proliferated more when incubated in a coculture system with Tconv from WT mice, compared to CD4 KO bone marrow cells cultured with Tconv in a transwell system (Fig. 5a, b). This coculture system again underlined the essential role of Tconv in inducing progenitor cell proliferation. Adding Tconv but not Treg cells enhanced proliferation of hematopoietic progenitors. Moreover, the proliferation did not differ in the presence or absence of serum from sham or MI mice, a result that indicates systemic factors released by MI are less important for inducing myelopoiesis (Suppl. Figure 9). Hence, consistent with the results presented in Fig. 2c, Tconv promote progenitor cell proliferation whereas Tregs have no impact on progenitors in the absence of Tconv (Suppl. Figure 9). As IFN-γ represents a prototypical cytokine secreted by activated conventional T-cells, we studied its effect on progenitor cell proliferation in our in vitro system. IFN-γ significantly elevated proliferation of HSCs, GMP/CMPs, and monocytes from CD4 knockout bone marrow (Fig. 6a, b). This indicates IFN-γ has a direct effect on progenitor cells and promotes myelopoiesis in the absence of additional remote factors and CD4^+^ T-cells.

Together, these experiments demonstrate that both cell–cell-contact-dependent mechanism and IFN-γ signalling are vital to the promoting effect CD4^+^ T-cells have on extramedullary myelopoiesis.

### Depletion of Tregs promotes proinflammatory differentiation of monocytes

**Fig. 7:**
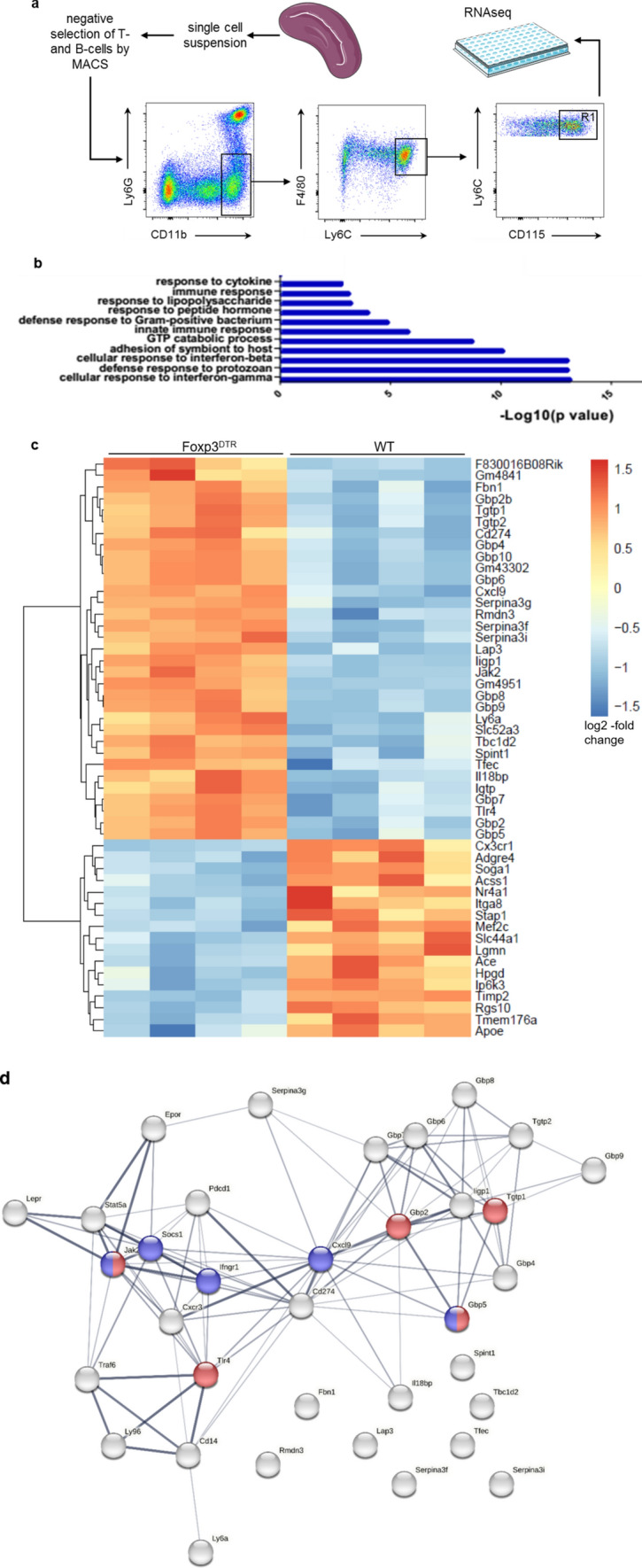
Transcriptome analysis of Ly6C^high^ monocytes in the spleens of WT and Foxp3^DTR^ mice 5 days after MI. **a** After CD3^+^ and CD19^+^ cells were depleted by MACS separation columns, Ly6C^high^ monocytes were sorted from R1 as CD11b^+^ Ly6G^−^ F4/80^+^ Ly6C^high^ CD115^+^ cells.** b** Gene ontology analysis showing clusters of upregulated genes in Ly6C^high^ monocytes of Foxp3^DTR^ vs. those of WT mice. **c** Cluster analysis of RNA sequencing results depicted as a heatmap (*n* = 4 animals per group; adjusted *p* < 0.05 for Foxp3^DTR^ vs. WT). Images were made using the Innate DB database (https://www.innatedb.com/) or the pheatmap package in R (https://www.rdocumentation.org/packages/pheatmap/versions/1.0.12/topics/ pheatmap). **d** Protein–protein interaction network model of the top upregulated genes. Red represents GO: 0034341“Response to interferone gamma”; Blue represents WP1253 “Type II Interferon signalling” (FDR < 0.0001 each)

Given the increased production of proinflammatory cytokines in the spleen in response to Treg depletion, we sought to determine if depleting CD4^+^ Foxp3^+^ T-cells might also impact monocyte differentiation in the spleen. Therefore, we sorted Ly6C^high^ monocytes from the spleen of WT mice after MI or sham surgery and diphtheria toxin-treated WT and Foxp3^DTR^ mice on day 5 after MI. Next, we performed RNA sequencing for transcriptome analysis (Fig. 7a). In WT animals, MI produced no significant changes in the gene expression of splenic monocytes, compared to animals that underwent sham surgery (Supplementary Table 1 lists the top up- and downregulated transcripts). However, the depletion of Tregs in Foxp3^DTR^ mice led to a significant shift in the transcriptome of splenic monocytes compared to that of monocytes from WT mice after MI (Supplementary Table 2). Notably, the transcriptional regulator Nr4a1, which regulates pro-healing macrophage differentiation from Ly6C^high^ monocytes in the infarcted myocardium [13], was amongst the top 50 downregulated transcripts in monocytes from Foxp3^DTR^ mice vs. WT mice after MI. Corresponding to the increased IFN-γ expression in splenic T-cells in Foxp3^DTR^ mice (Fig. 4c), the most significantly upregulated GO set of monocytes from Foxp3^DTR^ mice vs. WT mice after MI relates to transcripts associated with proinflammatory differentiation in response to IFN-γ stimulation (Fig. 7b,c). STRING-based protein–protein interaction network modelling (Fig. 7d) using these top upregulated genes revealed the IFN-γ receptor (*Ifngr1*) and the IFN-γ-induced chemokine CXCL9 as hubs. We further experimentally confirmed the relevance of IFN-γ for monocyte differentiation upon Treg deletion by analysing the surface-protein abundance of the chemokine receptor CX3CR1, which is amongst the most downregulated transcripts in Foxp3^DTR^ mice (Fig. 7c). Stimulating bone marrow cells ex vivo with IFN-γ downregulated CX3CR1 expression on monocytes (Suppl. Figure 10). Collectively, these data show that Tregs regulate monocyte differentiation in the spleen by mitigating the proinflammatory stimulus imposed by activated Tconv.

## Discussion

In the present study, we examine the role CD4^+^ T-cells play in regulating extramedullary myelopoiesis in response to MI. Our data show that the spleen is the major side of myelopoiesis on day 5 and splenic myelopoiesis requires the presence of Tconv which, via cell–cell-contact and paracrine mechanism, promote splenic myelopoiesis. Depletion of Treg cells enhances splenic myelopoiesis in response to MI and imposes a proinflammatory bias on the splenic monocytes. Tregs neither promote nor inhibit myelopoiesis by interacting with progenitors, but most likely indirectly by limiting Tconv activity.

Our previous experiments revealed that depleting Tregs leads to increased numbers of proinflammatory Ly6C^high^ monocytes in the myocardium and a concomitant downregulation of transcripts that promote healing in myocardial macrophages. We also showed that Tregs can interact with monocytes or monocyte-derived macrophages through soluble mediators to promote a pro-healing phenotype among macrophages in the myocardium [30]. In Treg-depleted mice, impaired systolic function was already evident on day 3 post-MI. The overall low frequency of regulatory T-cells in the myocardium raised the question of whether Tregs and monocytes interact at myelopoietic sites before entering the inflamed heart. Considering prior reports have demonstrated key roles for spleen-derived monocytes in infarct healing [20], myocardial remodelling [29], and atherosclerosis promotion after MI [4] as well as the induction of extramedullary but not bone marrow myelopoiesis in animals with genetic Treg deficiency [16], we primarily focussed our analysis on the spleen.

We found that splenic myelopoiesis in response to MI requires the presence of Tconv. Our data show that there is minimal splenic myelopoiesis in CD4KO mice after MI in vivo and Tconv promote progenitor cell proliferation in vitro. In addition, we found the relative and absolute numbers of Tregs in the spleen peak at day 5, when splenic myelopoiesis peaks. This indicates that Tregs likely have a regulatory effect in MI-induced splenic myelopoiesis. Accordingly, our data show that Tregs contribute to containing myelopoiesis in response to MI in vivo, though we were unable to elucidate the specific mechanism by which Tregs inhibit splenic pro-myelopoietic Tconv. It has previously been shown that in steady state, Tregs restrain T-cells that can produce myelopoiesis-promoting factors in the spleen by direct cell–cell-contact-dependent inhibitory actions [18, 27]. Treg-depleted animals exhibit spontaneous myelopoiesis in the spleen. Analogously, disinhibiting Tconv is likely the mechanism causing the enhanced myelopoiesis we observed after depleting Tregs. Hence, in our in vivo Treg-depletion experiments, the reduced tonic inhibition of pro-myelopoietic Tconv, due to the decreased numbers of endogenous Tregs, amplified MI-induced myelopoietic activity in the spleen. However, the in vivo experiments did not rule out the possibility that Treg depletion led to a systemic expansion of pro-myelopoietic factors, e.g. IL-1β [25], due to elevated inflammation in the myocardium. We therefore conducted in vitro experiments, which avoid any remote effects. The results indicate that enhanced myelopoiesis after Treg depletion relies not on signals from the infarcted heart but rather on the disinhibition of Tconv, which promote myelopoiesis by cell–cell contact and paracrine effects in vitro. Accordingly, we show in vivo that splenic Tconv produce myelopoietic cytokines after MI under Treg control. Correspondingly, Treg depletion in vivo increased the proportion of T-cells-producing IL-3, GM-CSF, IL-6, and IFN-γ. GM-CSF and IL-3 have been highlighted as myelopoiesis-promoting cytokines produced by splenic Tconv [18]. IFN-γ is a type II interferon primarily produced by T-cells and natural killer cells. Several studies on bone marrow hematopoiesis report that it exerts context-dependent either negative or positive regulatory effects [23]. In our in vitro culture system, IFN-γ promoted myelopoiesis. Considering the aforementioned different roles Tregs may play in regulating myelopoiesis in the spleen and bone marrow, we must acknowledge the limitation that, due to the low number of progenitors in the spleen, we were able to conduct the in vitro studies only with bone marrow cells. Collectively, our data show that in the absence of Tregs, splenic T-cells (including both CD4^+^ and CD8^+^ T-cells) overexpress several growth factors and cytokines that promote progenitor cell proliferation, which likely together enhance splenic myelopoesis.

In addition to progenitor cell proliferation, we found that Treg depletion induced a proinflammatory differentiation programme in splenic monocytes characterised by the expression of a set of IFN-γ-response transcripts. This corresponds to the finding that depleting Tregs induced elevated IFN-γ expression in the spleen. In comparison to diphtheria toxin-treated WT mice, the transcriptional profile of monocytes from Foxp3^DTR^ mice revealed the upregulation of genes, such as Gbp5 [11], associated with the IFN-γ -induced M1 macrophage phenotype; nevertheless, this transcriptional profile was distinct from that of in vivo-polarised M1-like macrophages [17]. Notably, depleting Tregs downregulated the expression of the transcription factor Nr4a1. This aligns with the reduced expression of pro-healing factors in myocardial macrophages from Treg-depleted animals [30]; Nr4a1 activity is necessary for Ly6C^high^ monocytes to acquire a Ly6C^low^ macrophage phenotype, which is a prerequisite for proper myocardial healing [13]. Our in vitro experiments further revealed that IFN-γ stimulation recapitulates the effect Treg depletion has on CX3CR1 expression. The chemokine receptor CX3CR1 inversely correlates with Ly6C and CCR2 surface expression and controls monocyte migration [19].

Collectively, we found that Tconv are necessary for induction of splenic myelopoiesis after MI. Endogenous Tregs curtail splenic myelopoiesis and prevent splenic monocytes from acquiring an IFN-γ-induced proinflammatory signature after acute MI, and these effects occur via mechanisms that include disinhibiting myelopoiesis-promoting T-cells. These novel data expand previous findings indicating that Tregs contribute to the local shift in monocyte-derived macrophage differentiation towards a pro-healing phenotype in the infarcted myocardium [15].

### Supplementary Information

Below is the link to the electronic supplementary material.

Supplementary file1 (PDF 932 KB)

## Data Availability

The RNA-Seq data discussed in this publication have been deposited in the NCBI’s Gene Expression Omnibus database and are accessible through GEO Series accession number GSE102817 (https://www.ncbi.nlm.nih.gov/geo/query/acc.cgi?acc=GSE102817). All other supporting data from this study are available within the article and the supplementary information or from the corresponding author upon reasonable request.
