# Supplementary material for: Role of CD4+ T-cells for regulating splenic myelopoiesis and monocyte differentiation after experimental myocardial infarction
Source: Basic Res Cardiol. 2024 Mar 4;119(2):261–75. doi: 10.1007/s00395-024-01035-3 (PMC11008073; doi:10.1007/s00395-024-01035-3)
Supplement: Supplementary file 1 — Supplementary file1 (PDF 932 KB) [file 395_2024_1035_MOESM1_ESM.pdf]

Role of CD4+ T-cells for regulating splenic myelopoiesis and monocyte differentiation after experimental myocardial infarction

Nadine Gladow[a,b]*, Claudia Hollmann[c], Johannes Weirather[d], Xin Ding[a,b], Matthias Burkard[a], Sabrina Uehlein[e], Richa Bharti[f], Konrad Förstner[g,h], Thomas Kerkau[c], Niklas Beyersdorf[c], Stefan Frantz[a,b], Gustavo Ramos[a,b], Ulrich Hofmann[a,b]

a Department of Internal Medicine I, University Clinic Würzburg, Würzburg, Germany

b Comprehensive Heart Failure Centre, University Clinic Würzburg, Würzburg, Germany

c Institute for Virology and Immunobiology, University of Würzburg, Würzburg, Germany

d MorphoSys AGPlanegg, Germany

e current affiliation: Department of Cardiology and Angiology, Hannover Medical School, Hannover, Germany

f TUM Campus Straubing for Biotechnology and Sustainability, Weihenstephan-Triesdorf University of Applied Sciences, Straubing, Germany

g ZB MED-Information Centre for Life Sciences, Cologne, Germany

h Faculty of Information Science and Communication Studies, Cologne University of Applied Sciences, Cologne, Germany

***Corresponding author:**
e-mail: Gladow_N@ukw.de

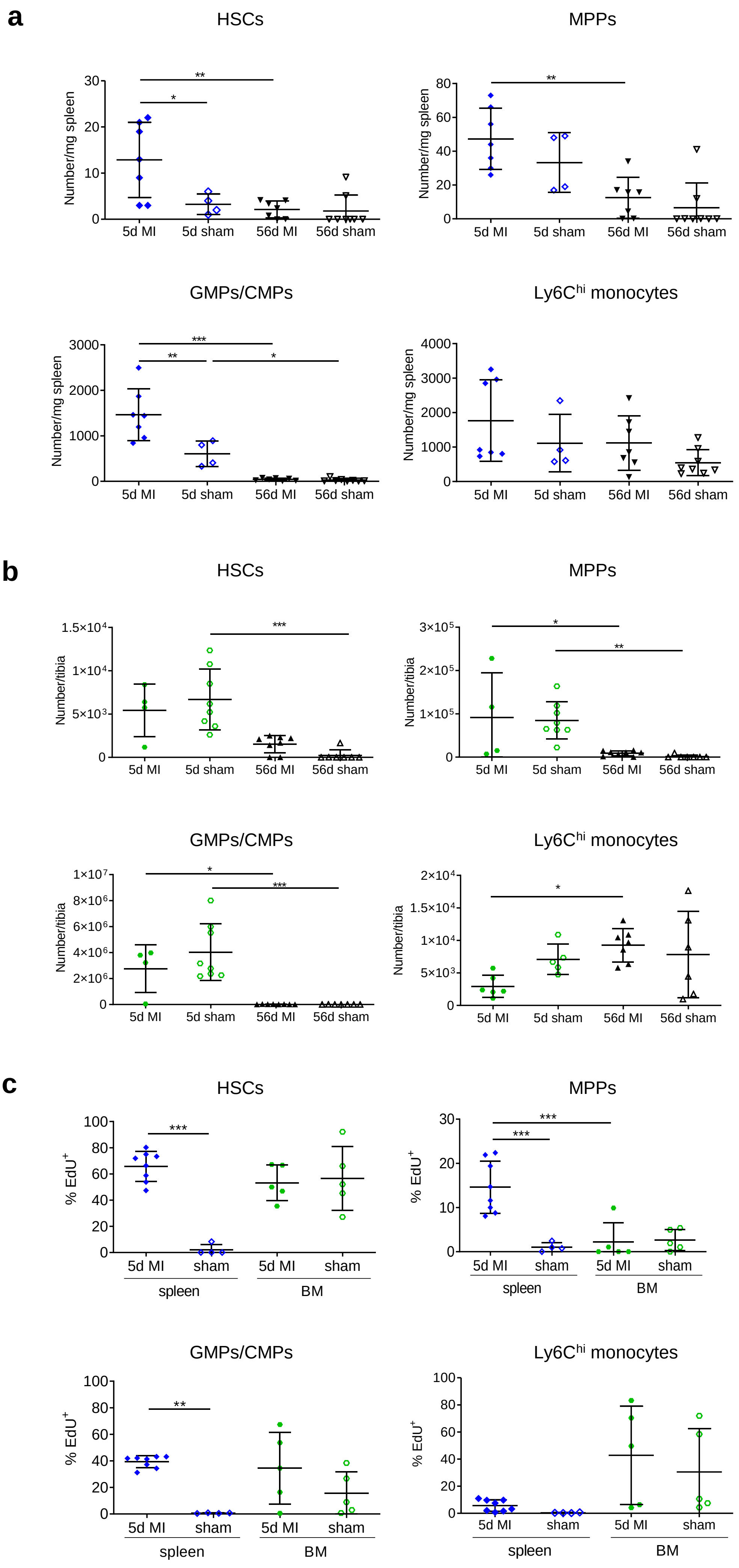

Supplementary Fig. 1 Quantification and proliferation analysis of precursor cells and monocytes in the spleen and bone marrow of wild type (WT) mice after MI or sham surgery. Absolute numbers of HSCs, MPPs, GMPs/CMPs, and monocytes in the spleen (**a**) and bone marrow (**b**) of WT mice 5 and 56 days after MI or sham operation. **c** In vivo proliferation of HSCs, MPPs, GMPs/CMPs, and monocytes in the spleen and BM 5 days after MI or sham operation. HSC = haematopoietic stem cell; MPP = multipotent progenitor; GMP = granulocyte-macrophage progenitor; CMP = common myeloid progenitor. Data are presented as the mean ± SD. *P<0.05, **P<0.01, ***P<0.001 (one-way ANOVA).

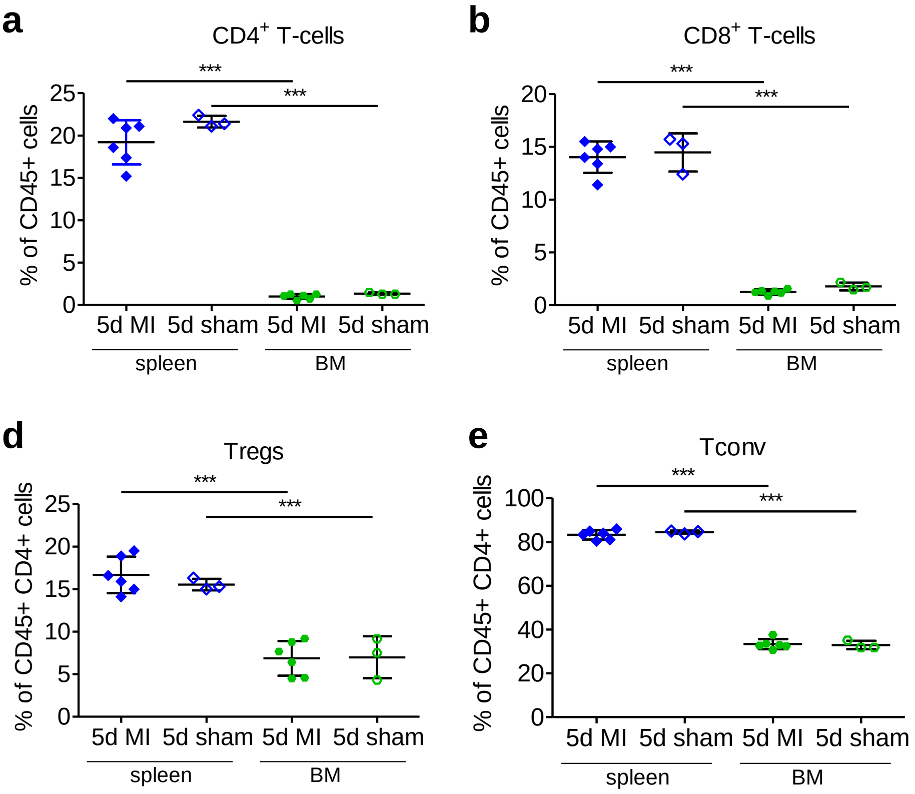

Supplementary Fig. 2 Frequencies of cells in the spleen and bone marrow (BM) 5 days after MI or sham operation. CD4+ (**a**), CD8+ (**b**) and CD19+ cells (**c**) among CD45+ cells in the spleen and bone marrow (BM) 5 days after MI (n=6 per group) or sham operation (n=3 per group). **d** and **e** showing the frequencies of Tregs or Tconvs among CD45+ CD4+ T-cells in the spleen and BM of WT mice 5 days after MI. **f** Ratio of Treg/Tconv frequencies in the spleen or bone marrow of infarcted and sham-operated mice 5 days after sham or MI surgery. BM= bone marrow; MI = myocardial infarction. Data are presented as the mean ± SD. **P<0.01, ***P<0.001 (one-way ANOVA).

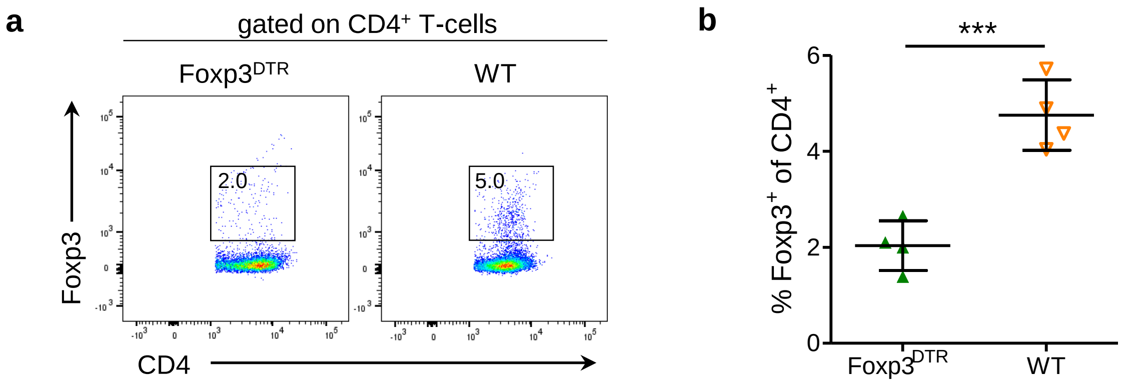

Supplementary Fig. 3 Foxp3⁺ T-cell depletion in the spleen. a Representative fluorescence-activated cell sorting plots and **b** quantitative analysis showing the frequency of Foxp3⁺ cells among CD4⁺ T-cells in the spleen of WT and T-cell depleted mice (Foxp3^{DTR}) 7 days after MI (n=4 per group). Data are mean ± SD. ***P<0.001 (**b** t-test)

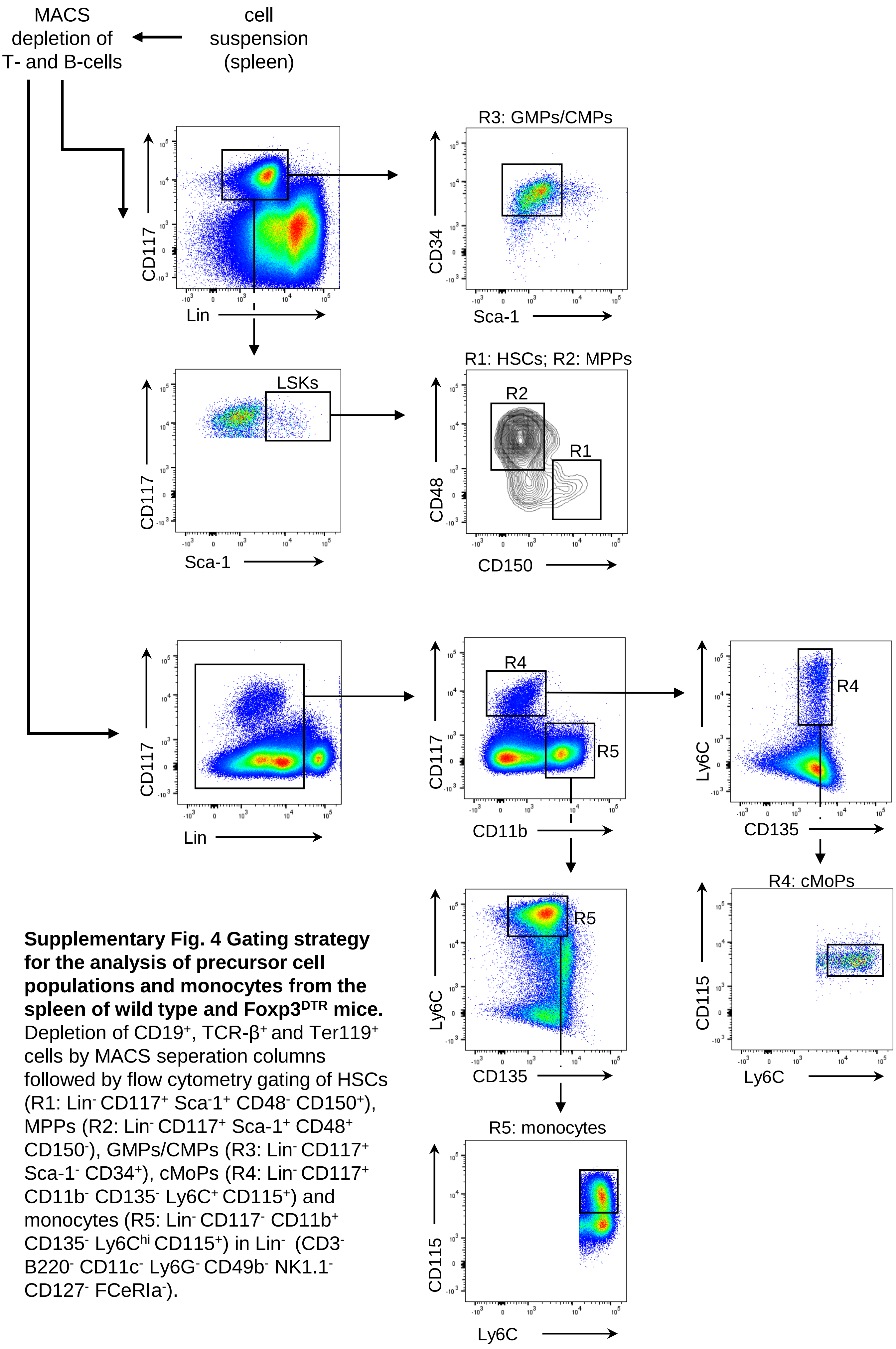

Supplementary Fig. 4 Gating strategy for the analysis of precursor cell populations and monocytes from the spleen of wild type and Foxp3^{DTR} mice. Depletion of CD19^{+}, TCR-β^{+} and Ter119^{+} cells by MACS seperation columns followed by flow cytometry gating of HSCs (R1: Lin^{-} CD117^{+} Sca-1^{+} CD48^{-} CD150^{+}), MPPs (R2: Lin^{-} CD117^{+} Sca-1^{+} CD48^{+} CD150^{-}), GMPs/CMPs (R3: Lin^{-} CD117^{+} Sca-1^{-} CD34^{+}), cMoPs (R4: Lin^{-} CD117^{+} CD11b^{-} CD135^{-} Ly6C^{+} CD115^{+}) and monocytes (R5: Lin^{-} CD117^{-} CD11b^{+} CD135^{-} Ly6C^{hi} CD115^{+}) in Lin^{-} (CD3^{-} B220^{-} CD11c^{-} Ly6G^{-} CD49b^{-} NK1.1^{-} CD127^{-} FCeRIa^{-}).

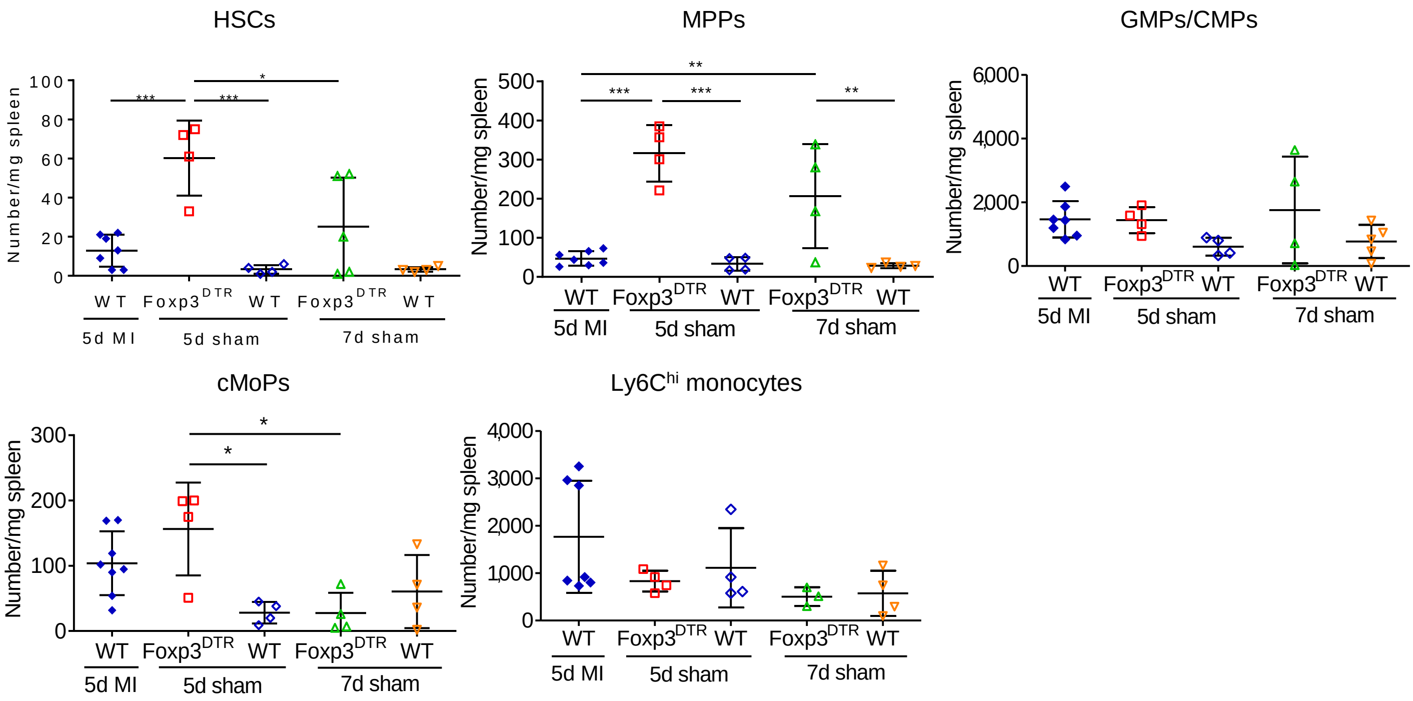

Supplementary Fig. 5 Quantification of the numbers of HSCs, MPPs, GMPs/CMPs, cMoPs and monocytes in the spleens of WT and Treg-depleted mice (Foxp3^{DTR}) at 5 and 7 days after MI or sham operation. HSC, haematopoietic stem cell; MPP, multipotent progenitor; GMP, granulocyte-monocyte progenitor; CMP, common myeloid progenitor; cMoP, common monocyte progenitor. Data are presented as the mean ± SD. *P<0.05, **P<0.01, ***P<0.001 (one-way ANOVA)

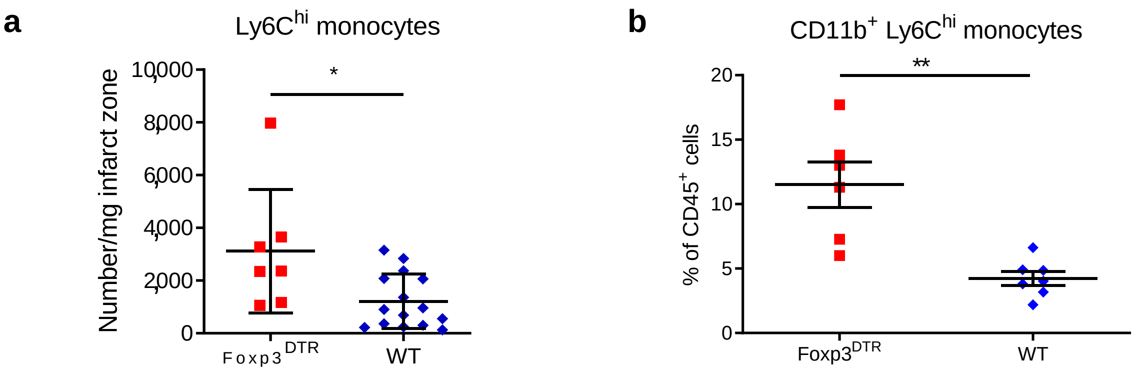

Supplementary Fig. 6 Number of Ly6C^{hi} monocytes in the myocardium (a) and blood (b) of Foxp3^{DTR} and wild type (WT) mice 7 days after myocardial infarction. Data are mean ± SD. *P<0.05, **>P<0.01 (U-test)

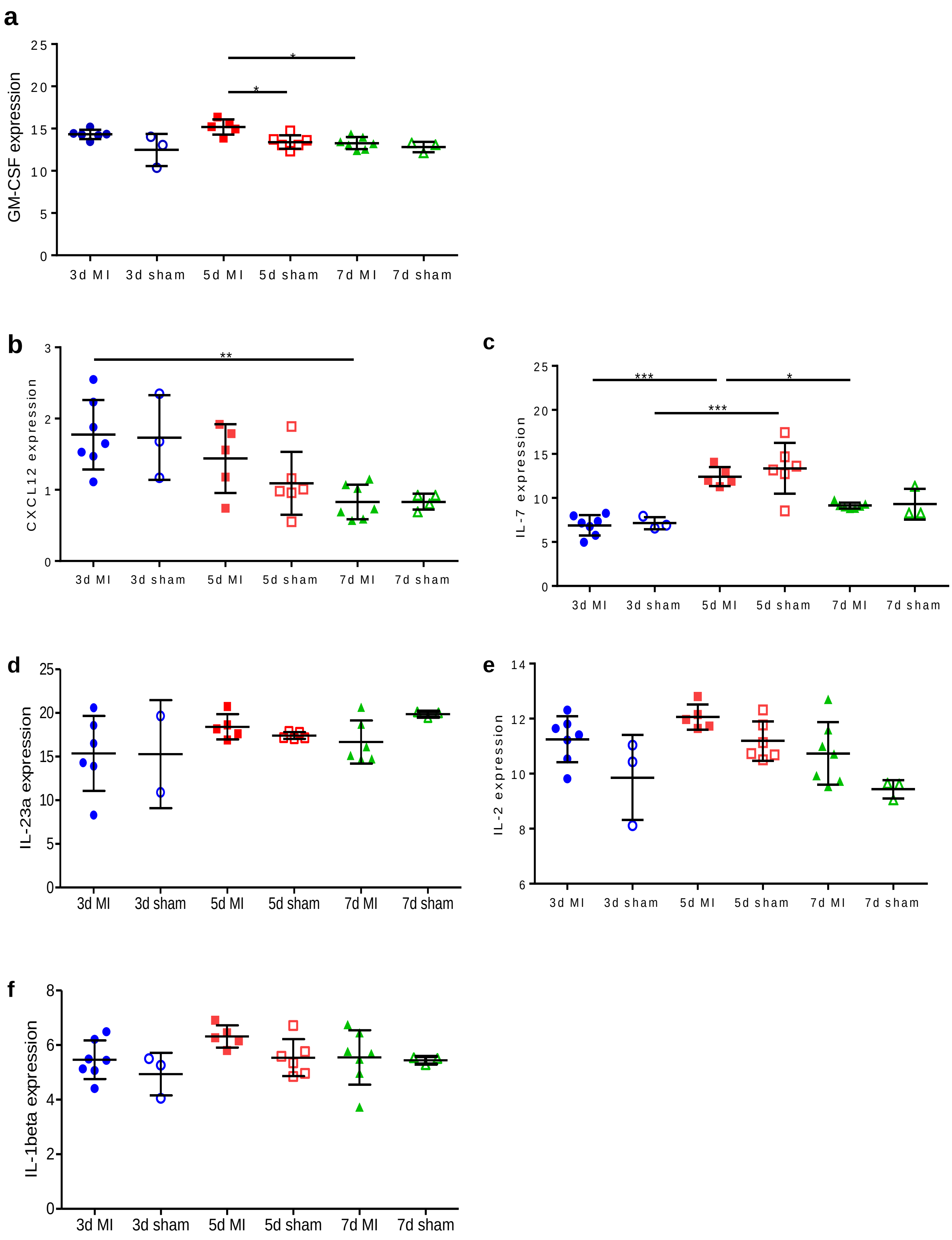

Supplementary Fig. 7 Cytokine expression in the spleen 3, 5 or 7 days after myocardial infarction (MI) or sham operation. Expression of GM-CSF (**a**) CXCL12 (**b**), IL-7 (**c**), IL23a (**d**), IL-2 (**e**) and IL-1beta (**f**) in the spleen of wild type mice after MI or sham surgery. Data are presented as the mean ± SD. *P<0.05, **P<0.01, ***P<0.001 (one-way ANOVA)

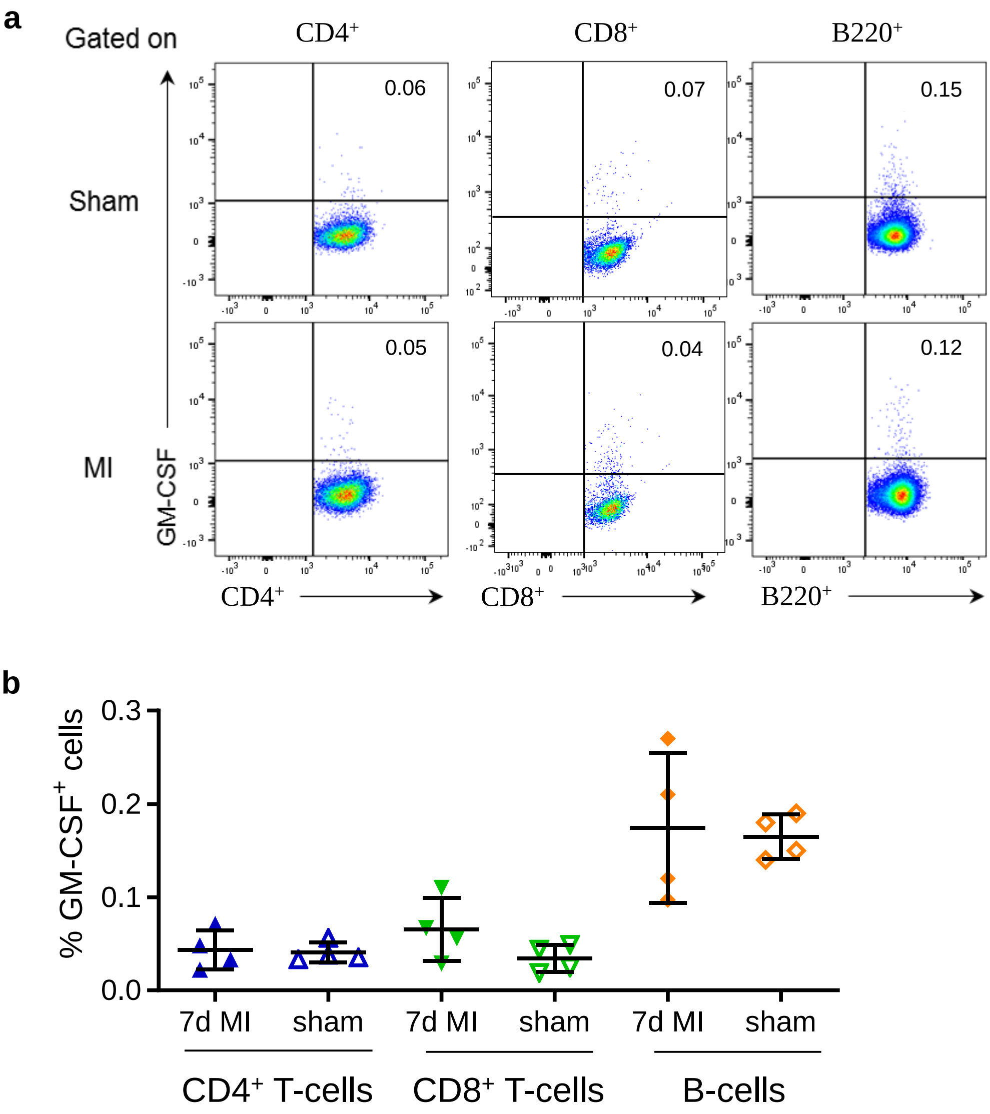

Supplementary Fig. 8 GM-CSF expression in the spleen 7 days after myocardial infarction (MI) or sham operation. Data are presented as the mean ± SD. not significant (t-test)

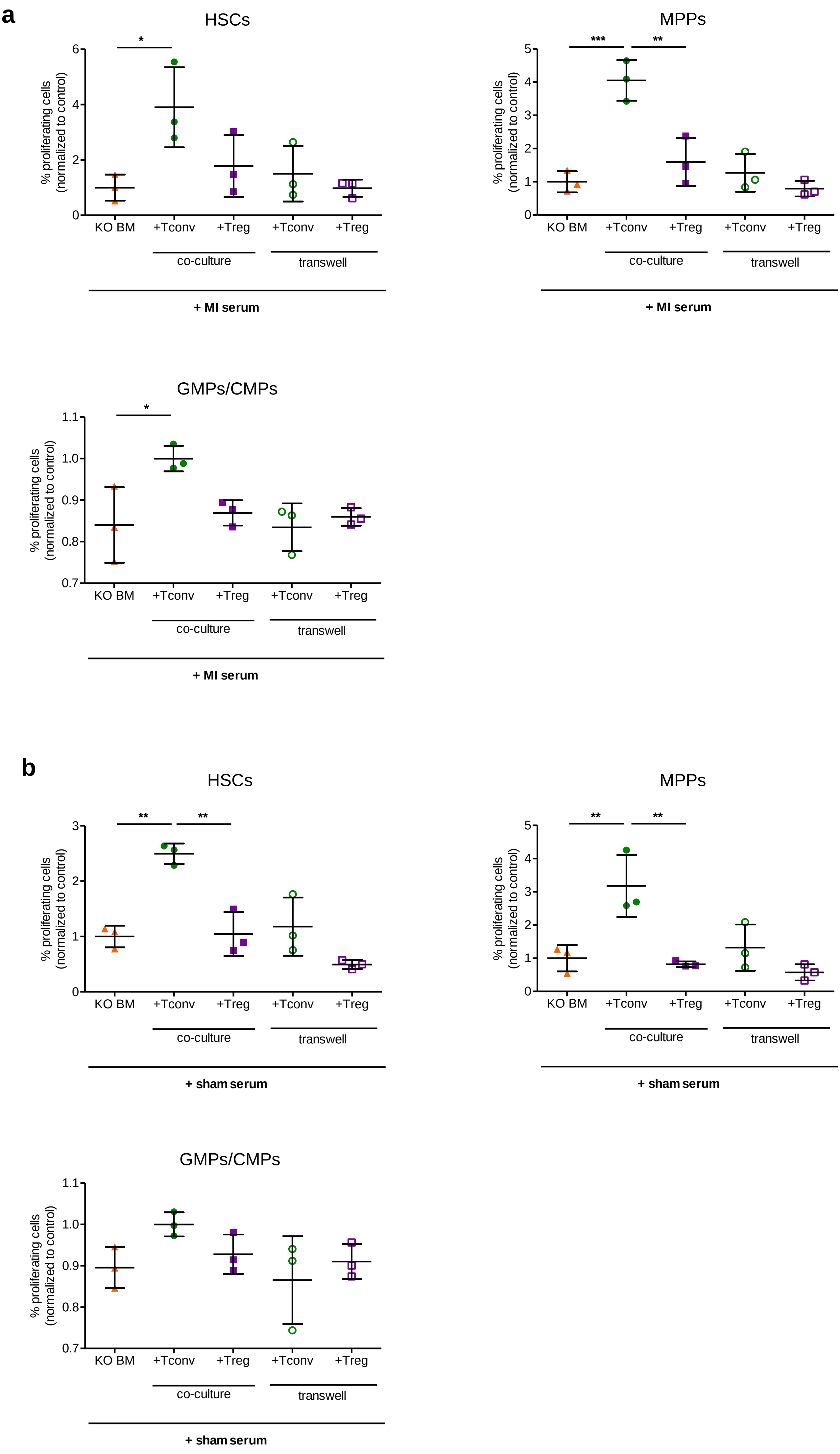

Supplementary Fig. 9 *In vitro* culture of Tregs or Tconv isolated bone marrow cells from CD4 KO mice. The proliferative activity of CD4KO BM cells in culture with serum from 5d MI (a) or sham (b) animals was measured by CFSE staining. Data are presented as the mean ± SD. *P<0.05, **P<0.01, ***P<0.001 (one-way ANOVA)

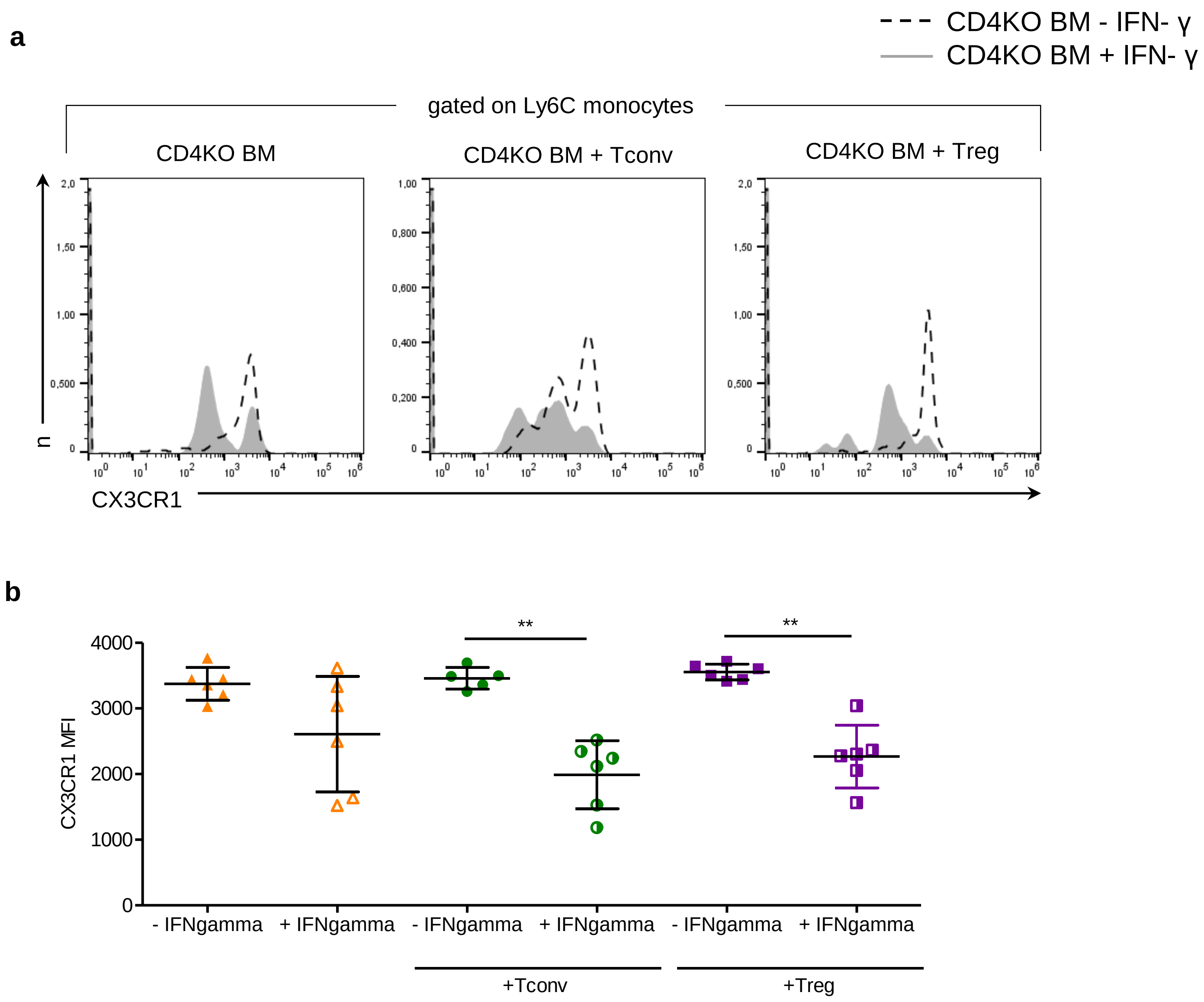

Supplementary Fig. 10 *In vitro* expression of CX3CR1$^+$ on monocytes.
a Representative fluorescence-activated cell sorting plots and **b** mean fluorescent intensity of CX3CR1$^+$ cells among Ly6C^{hi} monocytes from CD4KO BM cultured for 72h in presence or absence of IFN-gamma and Tconv or Treg cells isolated from WT spleens. Data are mean ± SD. **P<0.01 (**b** t-test)

Supplementary Table 1 Top 50 Upregulated (a) and downregulated (b) transcripts in splenic Ly6C^{high} monocytes (sorted as CD11b^{+} Ly6G^{-} F4/80^{+} Ly6C^{high} CD115^{+}) from WT mice 5 days after MI and sham surgery. Positive values mean upregulation in MI as compared to sham. (n=4, 5d MI; n=3, sham surgery).

a

Gene description	Gene symbol	log2 fold change	adjusted p-value
RAD18 E3 ubiquitin protein ligase	Rad18	0.67	1.00
PRKC, apoptosis, WT1, regulator	Pawr	0.57	1.00
rhotekin 2	Rtkn2	0.57	1.00
ankyrin repeat domain 37	Ankrd37	0.56	1.00
vascular endothelial growth factor C	Vegfc	0.55	1.00
membrane-spanning 4-domains, subfamily A, member 7	Ms4a7	0.54	1.00
NADH dehydrogenase subunit 6	ND6	0.54	1.00
predicted gene 5617	Gm5617	0.52	1.00
hairy and enhancer of split 7 (Drosophila)	Hes7	0.52	1.00
FERM domain containing 5	Frmd5	0.49	1.00
succinate dehydrogenase complex assembly factor 1	Sdhaf1	0.49	1.00
nudix (nucleoside diphosphate linked moiety X)-type motif 14	Nudt14	0.48	1.00
histone cluster 2, H2be	Hist2h2be	0.48	1.00
cytochrome c oxidase assembly factor 6	Coa6	0.48	1.00
NADH dehydrogenase (ubiquinone) 1 alpha subcomplex, assembly factor 4	Ndufaf4	0.47	1.00
NF-kappaB repressing factor	Nkrf	0.47	1.00
RIKEN cDNA 1700006E09 gene	1700006E09Rik	0.47	1.00
POU domain, class 4, transcription factor 1	Pou4f1	0.46	NA
IQ motif containing GTPase activating protein 3	Iqgap3	0.46	1.00
acid phosphatase 5, tartrate resistant	Acp5	0.46	1.00
isochorismatase domain containing 2b	Isoc2b	0.45	1.00
heat shock transcription factor family member 5	Hsf5	0.45	1.00
TBC1 domain family, member 30	Tbc1d30	0.45	1.00
barrier to autointegration factor 1	Banf1	0.44	1.00
B cell leukemia/lymphoma 2	Bcl2	0.44	1.00
minichromosome maintenance deficient 10 (S. cerevisiae)	Mcm10	0.44	1.00
elongator acetyltransferase complex subunit 6	Elp6	0.44	1.00
solute carrier family 36 (proton/amino acid symporter), member 3	Slc36a3	0.44	1.00
bora, aurora kinase A activator	Bora	0.44	1.00
predicted gene, 20594	Gm20594	0.43	1.00
ribonuclease H2, subunit B	Rnaseh2b	0.43	1.00
mitochondrial ribosomal protein L43	Mrpl43	0.43	1.00
c-mer proto-oncogene tyrosine kinase	Mertk	0.43	1.00
ribosomal protein, large, P1	Rplp1	0.43	1.00
zinc finger protein 1	Zfp1	0.43	1.00
transmembrane protein 203	Tmem203	0.43	1.00
histone cluster 1, H2ba	Hist1h2ba	0.43	1.00
ATP-binding cassette, sub-family A (ABC1), member 1	Abca1	0.43	1.00
RIKEN cDNA 3830403N18 gene	3830403N18Rik	0.43	1.00
cyclin-dependent kinase inhibitor 2C (p18, inhibits CDK4)	Cdkn2c	0.43	1.00
eosinophil-associated, ribonuclease A family, member 1	Ear1	0.43	1.00
POU domain, class 2, transcription factor 3	Pou2f3	0.42	1.00
apolipoprotein C-I	Apoc1	0.42	1.00
mutY homolog (E. coli)	Mutyh	0.42	1.00
phorbol-12-myristate-13-acetate-induced protein 1	Pmaip1	0.42	1.00
thyroid hormone receptor interactor 13	Trip13	0.42	1.00
proline rich 11	Prr11	0.41	1.00
budding uninhibited by benzimidazoles 1 homolog (S. cerevisiae)	Bub1	0.41	1.00
mitochondrial ribosomal protein L22	Mrpl22	0.41	1.00
fat storage-inducing transmembrane protein 2	Fitm2	0.41	1.00

The table continues on the next page.

Continuation of Supplementary Table 1 Top 50 Upregulated (a) and downregulated (b) transcripts in splenic Ly6C^high monocytes (sorted as CD11b^+ Ly6G^- F4/80^+ Ly6C^high CD115^+) from WT mice 5 days after MI and sham surgery. Positive values mean upregulation in MI as compared to sham. (n=4, 5d MI; n=3, sham surgery).

b

Gene description	Gene symbol	log 2 fold change	adjusted p-value
adhesion G protein-coupled receptor G1	Adgrg1	-0,75	1,00
high mobility group nucleosomal binding domain 3	Hmgn3	-0,68	1,00
GATA binding protein 2	Gata2	-0,66	1,00
epidermal growth factor receptor	Egfr	-0,64	1,00
vomeronasal 2, receptor 21	Vmn2r21	-0,62	1,00
transmembrane protease, serine 11f	Tmprss11f	-0,54	1,00
colony stimulating factor 1 (macrophage)	Csf1	-0,53	1,00
actin-binding LIM protein 2	Ablim2	-0,53	1,00
nephronophthisis 4 (juvenile) homolog (human)	Nphp4	-0,53	1,00
prickle homolog 3 (Drosophila)	Prickle3	-0,53	1,00
polymerase (RNA) III (DNA directed) polypeptide D	Polr3d	-0,52	1,00
sphingomyelin synthase 2	Sgms2	-0,52	1,00
sushi, nidogen and EGF-like domains 1	Sned1	-0,52	1,00
vomeronasal 2, receptor 22	Vmn2r22	-0,51	1,00
vomeronasal 2, receptor 20	Vmn2r20	-0,51	1,00
hemogen	Hemgn	-0,50	NA
polycystic kidney and hepatic disease 1-like 1	Pkhd1l1	-0,50	1,00
chromobox 6	Cbx6	-0,50	1,00
erythroblast membrane-associated protein	Ermap	-0,50	1,00
predicted gene 14322	Gm14322	-0,50	1,00
fibronectin type III and SPRY domain containing 2	Fsd2	-0,50	1,00
glycoprotein m6b	Gpm6b	-0,49	NA
carbohydrate (chondroitin 6/keratan) sulfotransferase 3	Chst3	-0,49	1,00
neuronal pentraxin chromo domain	Npcd	-0,49	1,00
chemokine (C-X-C motif) ligand 9	Cxcl9	-0,49	1,00
uncharacterized LOC101055912	LOC101055912	-0,49	1,00
serine peptidase inhibitor, Kazal type 2	Spink2	-0,48	1,00
target of myb1-like 1 (chicken)	Tom1l1	-0,48	1,00
metastasis suppressor 1	Mtss1	-0,48	1,00
peroxisomal biogenesis factor 11 alpha	Pex11a	-0,48	1,00
calcium channel, voltage-dependent, R type, alpha 1E subunit	Cacna1e	-0,48	NA
predicted gene 7120	Gm7120	-0,48	1,00
peptidyl arginine deiminase, type IV	Padi4	-0,47	1,00
enkurin, TRPC channel interacting protein	Enkur	-0,47	1,00
ADP-ribosylation factor-like 3	Arl3	-0,47	1,00
coiled-coil domain containing 7B	Ccdc7b	-0,46	1,00
tumor necrosis factor alpha induced protein 6	Tnfaip6	-0,46	1,00
par-6 family cell polarity regulator gamma	Pard6g	-0,46	NA
serine (or cysteine) peptidase inhibitor, clade C (antithrombin), member 1	Serpinc1	-0,46	1,00
RAB44, member RAS oncogene family	Rab44	-0,46	1,00
dematin actin binding protein	Dmtn	-0,46	1,00
Ets2 repressor factor	Erf	-0,45	1,00
family with sequence similarity 189, member A2	Fam189a2	-0,45	1,00
prostaglandin E synthase	Ptges	-0,45	1,00
PRA1 domain family 2	Praf2	-0,45	1,00
cytochrome P450, family 19, subfamily a, polypeptide 1	Cyp19a1	-0,45	1,00
solute carrier family 4 (anion exchanger), member 8	Slc4a8	-0,45	1,00
JAZF zinc finger 1	Jazf1	-0,45	1,00
sialic acid binding Ig-like lectin G	Siglecg	-0,45	1,00
family with sequence similarity 89, member A	Fam89a	-0,45	1,00

Supplementary Table 2 Top 50 up- (a) and downregulated (b) transcripts in splenic Ly6C^{high} monocytes (sorted as CD11b^{+} Ly6G^{-} F4/80^{+} Ly6C^{high} CD115^{+}) from Foxp3^{DTR} and WT mice 5 days after MI. Positive values mean upregulation in Foxp3^{DTR} as compared to WT (n=4 animals per group).

a

Gene description	Gene symbol	log2 fold change	Adjusted p-value
predicted gene 4951	Gm4951	5.20	3.16E-102
interferon inducible GTPase 1	Iigp1	4.93	1.25E-69
serine (or cysteine) peptidase inhibitor, clade A, member 3F	Serpina3f	3.18	2.64E-38
guanylate-binding protein 10	Gbp10	2.97	2.70E-36
guanylate binding protein 4	Gbp4	2.97	2.60E-30
guanylate binding protein 6	Gbp6	2.95	1.27E-32
RIKEN cDNA F830016B08 gene	F830016B08Rik	2.93	8.55E-18
chemokine (C-X-C motif) ligand 9	Cxcl9	2.83	1.67E-26
guanylate binding protein 2	Gbp2	2.81	2.95E-24
guanylate binding protein 2b	Gbp2b	2.66	1.07E-17
serine (or cysteine) peptidase inhibitor, clade A, member 3I	Serpina3i	2.60	3.45E-16
predicted gene 4841	Gm4841	2.57	1.00E-11
T cell specific GTPase 1	Tgtp1	2.51	1.14E-21
T cell specific GTPase 2	Tgtp2	2.50	3.42E-19
guanylate binding protein 5	Gbp5	2.31	1.87E-15
guanylate-binding protein 8	Gbp8	2.26	2.17E-41
cytokine inducible SH2-containing protein	Cish	2.17	8.62E-09
guanylate-binding protein 9	Gbp9	2.15	3.65E-38
serine (or cysteine) peptidase inhibitor, clade A, member 3N	Serpina3n	2.12	2.19E-07
serine protease inhibitor, Kunitz type 1	Spint1	2.08	5.42E-09
guanylate binding protein 7	Gbp7	1.98	1.32E-17
mast cell protease 8	Mcpt8	1.96	1.78E-06
matrix metallopeptidase 25	Mmp25	1.93	1.86E-08
5-hydroxytryptamine (serotonin) receptor 7	Htr7	1.87	3.36E-08
Der1-like domain family, member 3	Derl3	1.87	1.14E-05
lymphocyte antigen 6 complex, locus A	Ly6a	1.84	1.29E-12
protease, serine 34	Prss34	1.75	2.66E-05
fibrillin 1	Fbn1	1.73	1.71E-09
RIKEN cDNA 1500009L16 gene	1500009L16Rik	1.68	1.39E-04
interleukin 18 binding protein	Il18bp	1.61	2.69E-14
transcription factor EC	Tfec	1.57	9.18E-10
secreted and transmembrane 1A	Sectm1a	1.57	1.87E-05
CD274 antigen	Cd274	1.55	4.83E-11
interleukin 1 receptor-like 2	Il1rl2	1.54	4.22E-07
interleukin 12 receptor, beta 1	Il12rb1	1.51	3.96E-04
solute carrier family 30 (zinc transporter), member 4	Slc30a4	1.50	5.57E-08
guanylate binding protein 3	Gbp3	1.47	2.41E-08
interferon gamma induced GTPase	Igtp	1.47	1.67E-10
tumor necrosis factor, alpha-induced protein 8-like 3	Tnfaip8l3	1.46	5.66E-05
solute carrier organic anion transporter family, member 4C1	Slco4c1	1.41	6.35E-04
pyrophosphatase (inorganic) 1	Ppa1	1.39	1.03E-08
granzyme K	Gzmk	1.38	1.19E-03
leucine aminopeptidase 3	Lap3	1.38	4.85E-13
solute carrier protein family 52, member 3	Slc52a3	1.37	1.13E-09
cadherin 1	Cdh1	1.34	8.13E-06
interferon gamma inducible protein 47	Ifi47	1.33	7.94E-08
protein tyrosine phosphatase, non-receptor type 13	Ptpn13	1.33	1.13E-04
caspase 12	Casp12	1.32	5.52E-06
histamine receptor H2	Hrh2	1.32	4.34E-08
TBC1 domain family, member 2	Tbc1d2	1.32	7.37E-11

The table continues on the next page.

Continuation of Supplementary Table 2 Top 50 up- (a) and downregulated (b) transcripts in splenic Ly6C^{high} monocytes (sorted as CD11b^{+} Ly6G^{-} F4/80^{+} Ly6C^{high} CD115^{+}) from Foxp3^{DTR} and WT mice 5 days after MI. Positive values mean upregulation in Foxp3^{DTR} as compared to WT (n=4 animals per group).

b

Gene description	Gene symbol	log2 fold change	Adjusted p-value
integrin alpha 8	Itga8	-2.00	7.51E-12
hydroxyprostaglandin dehydrogenase 15 (NAD)	Hpgd	-1.95	4.83E-11
apolipoprotein E	Apoe	-1.86	2.00E-09
G protein-coupled receptor 83	Gpr83	-1.81	3.60E-06
suppressor of glucose, autophagy associated 1	Soga1	-1.69	2.97E-18
ATP-binding cassette, sub-family A (ABC1), member 8a	Abca8a	-1.64	5.24E-06
adhesion G protein-coupled receptor E4	Adgre4	-1.63	1.48E-13
platelet derived growth factor, B polypeptide	Pdgfb	-1.58	6.30E-06
predicted gene 1673	Gm1673	-1.54	8.67E-05
hairy and enhancer of split 1 (Drosophila)	Hes1	-1.54	3.51E-05
caspase recruitment domain family, member 10	Card10	-1.50	1.92E-07
chemokine (C-X3-C motif) receptor 1	Cx3cr1	-1.50	4.31E-15
angiotensin I converting enzyme (peptidyl-dipeptidase A) 1	Ace	-1.46	1.94E-12
transmembrane protein 176A	Tmem176a	-1.45	2.72E-14
inositol hexaphosphate kinase 3	Ip6k3	-1.43	3.78E-09
CD163 antigen	Cd163	-1.42	3.74E-04
adhesion G protein-coupled receptor L3	Adgrl3	-1.42	2.02E-05
Von Willebrand factor homolog	Vwf	-1.42	1.37E-08
acyl-CoA synthetase short-chain family member 1	Acss1	-1.41	5.00E-09
solute carrier family 46, member 3	Slc46a3	-1.41	1.24E-06
sciellin	Scel	-1.40	5.38E-08
signal transducing adaptor family member 1	Stap1	-1.40	2.97E-11
CD209a antigen	Cd209a	-1.38	1.32E-03
FMS-like tyrosine kinase 3	Flt3	-1.37	1.73E-04
transmembrane protein 176B	Tmem176b	-1.36	1.31E-06
thrombospondin 1	Thbs1	-1.35	7.36E-05
wingless-type MMTV integration site family, member 11	Wnt11	-1.33	NA
myosin XVIIIb	Myo18b	-1.31	9.08E-06
ring finger protein 150	Rnf150	-1.27	2.29E-04
nuclear receptor subfamily 4, group A, member 1	Nr4a1	-1.27	5.71E-10
ATP-binding cassette, sub-family A (ABC1), member 6	Abca6	-1.27	5.84E-04
whirlin	Whrn	-1.27	1.56E-05
Spi-C transcription factor (Spi-1/PU.1 related)	Spic	-1.26	7.14E-04
pleckstrin homology domain containing, family H (with MyTH4 domain) member 3	Plekhh3	-1.24	4.63E-04
collagen, type IV, alpha 1	Col4a1	-1.24	3.49E-04
RIKEN cDNA A930011G23 gene	A930011G23Rik	-1.22	2.44E-03
tissue inhibitor of metalloproteinase 2	Timp2	-1.22	6.92E-21
sphingosine-1-phosphate receptor 5	S1pr5	-1.21	9.51E-07
potassium voltage-gated channel, subfamily Q, member 3	Kcnq3	-1.21	4.53E-04
frizzled homolog 1 (Drosophila)	Fzd1	-1.19	9.03E-03
POU domain, class 4, transcription factor 1	Pou4f1	-1.19	NA
arylsulfatase i	Arsi	-1.19	6.23E-04
patched domain containing 1	Ptchd1	-1.18	NA
retrotransposon gag domain containing 4	Rgag4	-1.17	2.65E-06
calcium channel, voltage-dependent, alpha 1F subunit	Cacna1f	-1.17	6.24E-03
T cell acute lymphocytic leukemia 2	Tal2	-1.16	NA
pyrimidinergic receptor P2Y, G-protein coupled, 6	P2ry6	-1.15	3.80E-06
ATP-binding cassette, sub-family A (ABC1), member 1	Abca1	-1.15	1.54E-04
nuclear receptor subfamily 1, group D, member 2	Nr1d2	-1.15	8.56E-06
cadherin 22	Cdh22	-1.14	4.43E-03
